# Genomic Analysis of the 1-Aminocyclopropane-1-Carboxylate Deaminase-Producing *Pseudomonas thivervalensis* SC5 Reveals Its Multifaceted Roles in Soil and in Beneficial Interactions With Plants

**DOI:** 10.3389/fmicb.2021.752288

**Published:** 2021-09-30

**Authors:** Francisco X. Nascimento, Paola Urón, Bernard R. Glick, Admir Giachini, Márcio J. Rossi

**Affiliations:** ^1^iBET, Instituto de Biologia Experimental e Tecnológica, Oeiras, Portugal; ^2^Laboratório de Microbiologia e Bioprocessos, Departamento de Microbiologia, Universidade Federal de Santa Catarina, Florianópolis, Brazil; ^3^Department of Biology, University of Waterloo, Waterloo, ON, Canada

**Keywords:** *Pseudomonas*, plant-growth-promoting bacteria, 1-aminocyclopropane-1-carboxylate deaminase, genomics, plant-microbe interaction

## Abstract

Beneficial 1-aminocyclopropane-1-carboxylate (ACC) deaminase-producing bacteria promote plant growth and stress resistance, constituting a sustainable alternative to the excessive use of chemicals in agriculture. In this work, the increased plant growth promotion activity of the ACC deaminase-producing *Pseudomonas thivervalensis* SC5, its ability to limit the growth of phytopathogens, and the genomics behind these important properties are described in detail. *P. thivervalensis* SC5 displayed several active plant growth promotion traits and significantly increased cucumber plant growth and resistance against salt stress (100mmol/L NaCl) under greenhouse conditions. Strain SC5 also limited the *in vitro* growth of the pathogens *Botrytis cinerea* and *Pseudomonas syringae* DC3000 indicating active biological control activities. Comprehensive analysis revealed that *P. thivervalensis* SC5 genome is rich in genetic elements involved in nutrient acquisition (N, P, S, and Fe); osmotic stress tolerance (e.g., glycine-betaine, trehalose, and ectoine biosynthesis); motility, chemotaxis and attachment to plant tissues; root exudate metabolism including the modulation of plant phenolics (e.g., hydroxycinnamic acids), lignin, and flavonoids (e.g., quercetin); resistance against plant defenses (e.g., reactive oxygens species-ROS); plant hormone modulation (e.g., ethylene, auxins, cytokinins, and salicylic acid), and bacterial and fungal phytopathogen antagonistic traits (e.g., 2,4-diacetylphloroglucinol, HCN, a fragin-like non ribosomal peptide, bacteriocins, a lantipeptide, and quorum-quenching activities), bringing detailed insights into the action of this versatile plant-growth-promoting bacterium. Ultimately, the combination of both increased plant growth promotion/protection and biological control abilities makes *P. thivervalensis* SC5 a prime candidate for its development as a biofertilizer/biostimulant/biocontrol product. The genomic analysis of this bacterium brings new insights into the functioning of *Pseudomonas* and their role in beneficial plant-microbe interactions.

## Introduction

The increasing demand for sustainable agricultural practices creates the need for the development of novel strategies to promote plant growth and stress resistance without resorting to harmful chemical fertilizers and pesticides. One alternative to such polluting chemical compounds resides in the use of plant-growth-promoting bacteria (PGPB) that are able to efficiently promote plant growth and health (Glick, 1995; de Souza et al., 2015). These beneficial plant-associated bacteria are naturally found in soils and plant tissues, and are known to help plants overcome numerous growth limitations and stressful conditions, being of great importance for agricultural and biotechnological applications (Glick, 2012).

One of the most important mechanisms employed by PGPB involves the expression of the enzyme 1-aminocyclopropane-1-carboxylate (ACC) deaminase and the subsequent modulation of the plant hormone ethylene through the catabolism of its direct precursor, ACC (Glick et al., 1998; Glick, 2014). Ethylene is one of the most important plant hormones regulating plant growth and development, being involved in multiple physiological and developmental processes of plants (Van de Poel et al., 2015; Dubois et al., 2018), as well as in the regulation of plant-microbe interactions (Guinel, 2015; Nascimento et al., 2018a). Moreover, ethylene is also involved in plant responses to stress conditions, including those induced by biotic (e.g., pathogens, insects, and nematodes) and abiotic (e.g., salinity, low nutrient availability, low pH, heavy metals, and organic contaminants) stress factors (Lund et al., 1998; Broekaert et al., 2006; Gamalero and Glick, 2015; Tao et al., 2015; Keunen et al., 2016).

Bacteria presenting ACC deaminase activity decrease the deleterious levels of biotic and abiotic stress-induced ACC and ethylene that inhibit plant growth (Ma et al., 2004; Mayak et al., 2004; Belimov et al., 2009; Nascimento et al., 2013; Ali et al., 2014; Jaemsaeng et al., 2018). The beneficial and protective effect of ACC deaminase-producing bacteria often leads to the increased selection of these bacteria in bacterial communities associated with stressed plants (Timmusk et al., 2011; Croes et al., 2013; Truyens et al., 2013). Moreover, ACC deaminase genes (*acdS*) are positively selected in rhizobial symbionts associated with several leguminous plants worldwide (Nascimento et al., 2018b), further emphasizing the importance of this enzyme in beneficial plant-microbe interactions. Altogether, this data indicates that obtaining and selecting efficient ACC deaminase-producing bacteria is crucial for the development of successful biofertilizers with both increased plant growth promotion potential and an increased ability to protect plants from biotic and abiotic stress.

In a recent study, the direct isolation of several ACC deaminase-producing bacteria from various plants and locations was reported (Nascimento et al., 2019). One of the isolated strains, *Pseudomonas thivervalensis* SC5, presented very high levels of ACC deaminase activity suggesting an increased plant growth promotion potential (Nascimento et al., 2019).

The present work deals with the characterization, plant growth promoting activities and genomic properties of *P. thivervalensis* SC5.

## Materials and Methods

### *Pseudomonas thivervalensis* SC5 Characterization

*Pseudomonas thivervalensis* SC5 was routinely grown in Tryptic Soy Broth (TSB, Casvi, Brazil) at 28°C, 150rpm, overnight. The fresh bacterial suspensions were then used in the biochemical characterization assays, which were conducted in duplicate.

Briefly, strain SC5 was qualitatively tested for the ability to synthesize extracellular enzymes such as protease (in skim milk agar, Himedia), lipase (in rodamine B+ olive oil agar, Kouker and Jaeger, 1987), esterase (in Tributyrin agar, Himedia), amylase (Nutrient Broth supplemented with 5g/l starch; Gopinath et al., 2017), and cellulase (carboxymethylcellulose-CMC-agar; Kasana et al., 2008; Vicente et al., 2012). The strain was also tested for phosphate (PO_4_) and zinc oxide (ZnO) solubilization (de Freitas et al., 1997; Sharma et al., 2011); siderophores production in Chrome Azurol-S Agar (Schwyn and Neilands, 1987) and in King’s B agar (*Pseudomonas* agar F, Himedia; presence of fluorescence); amino acid decarboxylase/polyamines production using Moeller Decarboxylase Broth supplemented with 2% L-Arginine, or L-Lysine, or L-Ornithine (Himedia); the biosynthesis of ammonia in proteose peptone broth using the Nessler reagent as indicator (Marques et al., 2010); the ability to produce H_2_S and indole, as well as the ability to present motility were tested using Sulfide Indole Motility (SIM) media (Sigma-Aldrich); the biosynthesis of indole-3-acetic acid (IAA) in TSB supplemented with 500μg/ml of tryptophan and posterior detection using the Salkowski reagent (Glickmann and Dessaux, 1995); nitrate/nitrite reduction (Buxton, 2011); resistance to high salinity (growth in TSB supplemented with 3 and 5% w/v NaCl); resistance to antibiotics (growth in TSB supplemented with ampicillin-100μg/ml, chloramphenicol-30μg/ml, kanamycin-50μg/ml, and tetracycline-15μg/ml); and the ability to use 4-aminobutyrate (GABA), KNO_3_, KNO_2_, and phytohormones [IAA, benzoic acid (BA), salicylic acid (SA), abscisic acid (ABA), jasmonic acid (JA), gibberellic acid-GA3-] as sole carbon/nitrogen sources, as previously described (Nascimento et al., 2019).

### Plant Growth Promotion Assays

#### Cucumber Plant Growth Promotion Assay Under Laboratory Conditions

Commercial cucumber “Straight eight” (*Cucumis sativus*) seeds (Isla, Brazil) were surface disinfected by immersion in 70% ethanol for 1min, followed by 10min in 1% sodium hypochlorite. The seeds were then rinsed five times in sterile deionized water. The seeds were distributed into Petri dishes containing 1% water agar and incubated in the dark at 25°C for 3days. Each germinated seed was sown in a 300ml (volume) pot filled with sterilized vermiculite and sand in a 1:1 (v/v) ratio. The experiment consisted of two treatments: the non-inoculated control, and SC5 inoculation. The inoculation consisted of soaking the germinating seeds for 1h in an overnight grown bacterial suspension (TSB, 28°C, 150rpm) adjusted to OD_600_=0.3 in sterile 0.03M MgSO_4_, followed by the direct application of 3ml of a bacterial suspension (OD_600_=0.3) into the root-shoot junction of germinated plants. Six plants were used for each treatment. The assay was conducted under laboratory conditions with an average temperature of 25°C and a 12h photoperiod. The plants received 10ml Hoagland’s nutrient solution (Hoagland and Arnon, 1950) whenever necessary, and were harvested 3weeks after inoculation. The plant roots and shoots were excised and dried for 3days at 60°C in order to record dry weights on an analytical scale.

#### Cucumber Plant Growth Promotion Assay Under Normal and Salt Stress Conditions in the Greenhouse

Salt stress is one the major inducers of ACC and ethylene production by several plant species, which ultimately leads to plant growth inhibition (root and shoot development, overall biomass; Tao et al., 2015). The fine-tuning of ACC and ethylene levels under salt stress conditions is therefore of importance for increased plant salt stress resistance (Riyazuddin et al., 2020). Since *P. thivervalensis* SC5 presented both ACC deaminase activity, salt stress resistance and overall plant growth promotion abilities, we hypothesized that this strain could be useful for increasing cucumber plant salt stress resistance. Cucumber is known for its high sensitivity to salt stress (Gamalero et al., 2010). Based on this hypothesis, *P. thivervalensis* SC5 was tested for its ability to help salt-sensitive cucumber plants to overcome some of the deleterious effects of salt stress (100mmol/l NaCl).

The preparation of the experiment (seed germination, inoculation, and pots) was conducted as described above (see Section “Cucumber Plant Growth Promotion Assay UnderLaboratory Conditions”).

The experiment consisted of four treatments: non-inoculated control (normal conditions); *P. thivervalensis* SC5 inoculation (normal conditions); non-inoculated control+100mmol/L NaCl (salt stress conditions); *P. thivervalensis* SC5 inoculation +100mmol/L NaCl (salt stress conditions). Six plants were used for each treatment. In the initial 5days of growth the plants received 5ml Hoagland’s nutrient solution whenever necessary (dried top layer of vermiculite and sand). After this period, stress conditions were imposed (to the respective salt receiving treatments) by applying 10ml Hoagland’s nutrient solution containing 100mmol/L NaCl 4days per week. The non-inoculated control and *P. thivervalensis* SC5 inoculation treatments (normal conditions) received 10ml Hoagland’s nutrient solution 4days per week.

The greenhouse experiment (average temperature 26.9°C) was conducted in the Universidade Federal de Santa Catarina, Florianópolis, Brazil. About 3weeks after sowing, cucumber plants were harvested, washed with tap water, and root and shoot lengths (SLs) were measured. The plants were then dried at 60°C and root and shoot dry weights were measured.

### Statistical Analysis

The SPSS Statistics v. 26 software (SPSS, IBM Company, United States) was used to perform a statistical analysis, which was conducted using an ANOVA and means comparison using Tukey’s test.

### Phytopathogen Antagonistic Activities

Strain SC5 antagonistic activities were tested against the fungal pathogen *Botrytis cinerea* and the bacterial pathogen *Pseudomonas syringae* DC3000 as previously described (Nascimento et al., 2016). The fungal antagonistic activities were detected by the inability of the fungal strain to grow over SC5 spots in potato dextrose agar plates. The bacterial antagonistic activities were detected by the inability of *P. syringae* DC3000 to grow in the presence of strain SC5.

### Genome Sequencing and Analysis

The SC5 DNA library was constructed using Illumina TruSeq DNA Nano kit (automated) and sequenced in the Illumina MiSeq platform using the MiSeq V3 reagent kit (2×300bp).

The complete 6,592,350bp genome sequence of strain SC5 is a scaffold of 37 contigs (N50=313,629bp), which were generated by a total of 1,480,168 reads (coverage of 67X) assembled using SOAPdenovo v2.04 (Luo et al., 2012), and constructed based on a guided assembly against the complete genome sequence of *P. thivervalensis* BS3779=DSM 13194^T^=LMG 21626^T^ (NZ_LT629691.1) using Mauve (Darling et al., 2004). The contigs were joined by introducing 100 Ns in the assembly gap regions as indicated in the NCBI submission guidelines. The genome sequence of *P. thivervalensis* SC5 is available in the NCBI^1^ under the accession number CP022201.1.

The genome annotation was performed using the NCBI Prokaryotic annotation pipeline (Angiuoli et al., 2008). Functional genome annotation and analysis were conducted as described elsewhere (Nascimento et al., 2020). Genome circular view was created using CGview (Grant and Stothard, 2008).

Average nucleotide identity (ANI) was calculated using PyANI and the ANIb method (Pritchard et al., 2016).

## Results and Discussion

### Characterization and Plant Growth Promotion Properties of *P. thivervalensis* SC5

A general characterization of *P. thivervalensis* SC5 was conducted and is presented in Table 1. Strain SC5 is a Gram-negative, rod shaped, motile, and non-sporulating bacterium that can grow in temperatures ranging from 7 to 30°C and in NaCl concentrations up to 5%. It is resistant to ampicillin (100μg/ml) and chloramphenicol (30μg/ml) but found to be sensitive to kanamycin (50μg/ml) or tetracycline (15μg/ml). Strain SC5 produced siderophores, including a pyoverdine-like pigment (presents fluorescence when grown in King’s B medium); solubilized PO_4_ and ZnO; produced polyamines when supplemented with arginine but not lysine or ornithine; produced low levels of IAA (or IAA-like compounds), ~4μg/ml, in the presence of exogenous tryptophan (500μg/ml); and reduced nitrate to N_2_ and ammonia, which is consistent with its ability to use KNO_3_ and KNO_2_ as sole nitrogen sources. This bacterium is positive for the extracellular lytic enzyme lipase but negative for esterase, amylase, cellulase, and protease under the tested conditions. Strain SC5 is not able to use IAA, salicylic acid (SA), benzoic acid (BA), abscisic acid (ABA), jasmonic acid (JA), and gibberellins (GA) as sole carbon sources. On the other hand, it presented the ability to consume 4-aminobutyrate (GABA).

**Table 1:** Phenotypic characterization of *P. thivervalensis* SC5.

Traits	Characteristics
Antibiotic resistance	(+) Ampicillin (100μg/ml),
	(+) Chloramphenicol (30μg/ml)
	(−) Kanamycin (50μg/ml)
	(−) Tetracycline (15μg/ml)
Siderophore production	(+) Chrome-Azurol-S agar;
	(+) fluorescence when grown in King’s B medium (pyoverdine-like siderophore)
PO_4_ solubilization	(+) in PDYA-CaP agar
ZnO solubilization	(+) in Tris Agar+ ZnO
Polyamine biosynthesis from:	(+) arginine
	(−) lysine
	(−) ornithine
IAA biosynthesis	3.89±0.05μg/ml
ACC deaminase activity^*^	18.592±0.203μmol α-ketobutyrate/mg protein/h
Extracellular Lipase activity	(+) in Rhodamine B+ olive oil agar
Extracellular Esterase activity	(−) in Tributyrin Agar
Extracellular protease activity	(−) in Skimmed milk agar
Extracellular cellulase activity	(−) in CMC agar
Extracellular amylase activity	(−) in NB+starch agar
Ability to grow in M9 minimal medium supplemented with:	(+) KNO_3_ (N source)
	(+) KNO_2_ (N source)
	(+) GABA (N source)
	(−) IAA (C source)
	(−) SA (C source)
	(−) BA (C source)
	(−) ABA (C source)
	(−) GA_3_ (C source)

(+)- positive (presence of halo, or growth, when applicable).

(−)- negative (absence of halo, or growth, when applicable).

* Nascimento et al. (2019).

Laboratory experiments indicated that the inoculation of *P. thivervalensis* SC5 led to a significant increase in cucumber plant growth (Supplementary Figure S1) when compared to the non-inoculated control plants, 3weeks after inoculation, confirming the plant growth promoting properties of strain SC5.

These results indicate that the ACC deaminase-producing *P. thivervalensis* SC5 has several active plant growth promotion properties and acts as an active PGPB, which is consistent with previous reports showing the increased plant growth promotion abilities of ACC deaminase-producing *Pseudomonas* strains (Glick, 1995; Rashid et al., 2012).

### *Pseudomonas thivervalensis* SC5 Promotes Cucumber Growth Under Normal and Salt Stress Conditions

**Figure 1:**
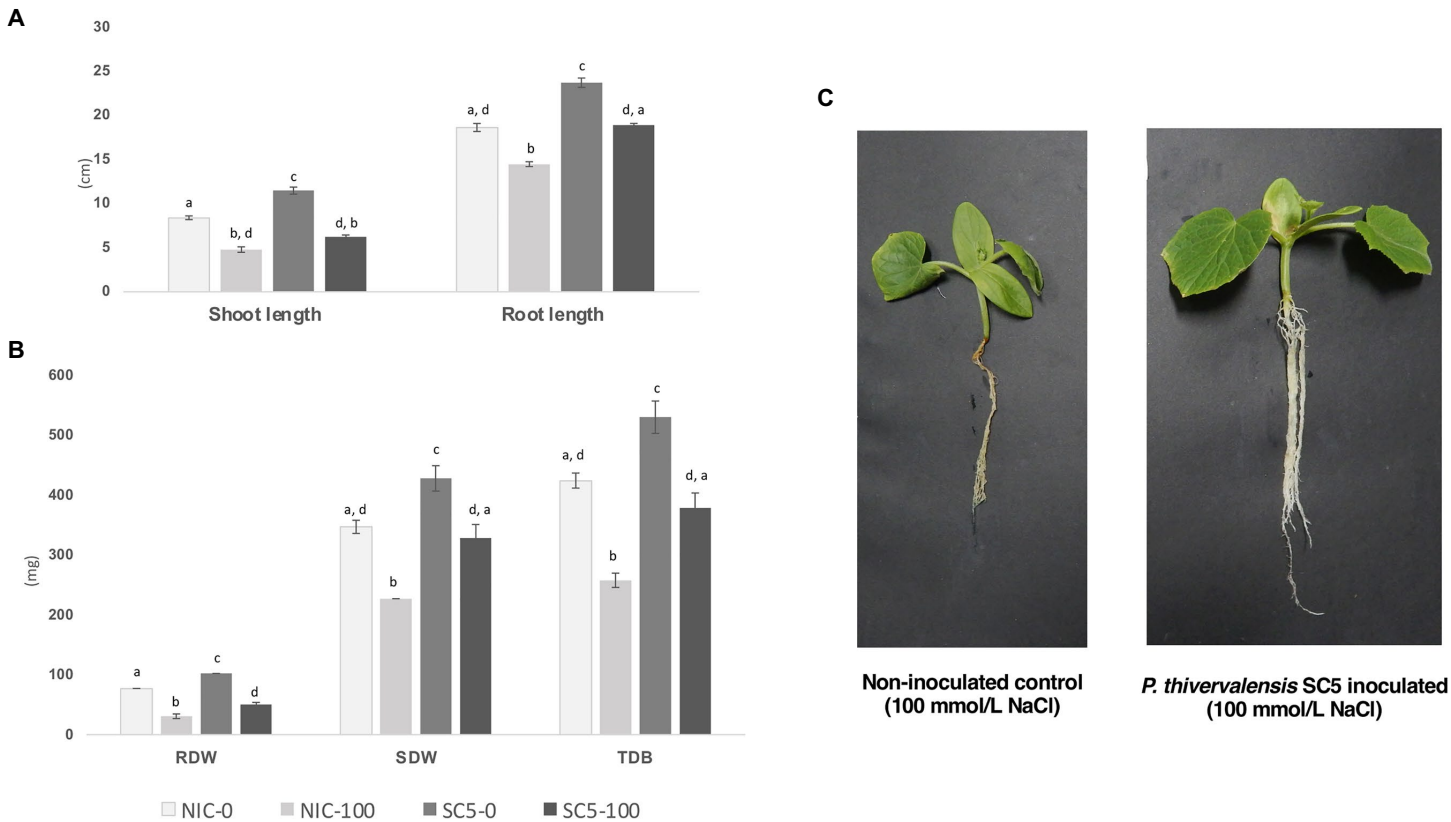
Results obtained from a cucumber plant growth promotion assay performed under greenhouse conditions, 3weeks after inoculation, six replicates. **(A)** Shoot and Root lengths (cm) of non-inoculated control and *Pseudomonas thivervalensis* SC5 inoculated plants under normal and salt stress conditions; **(B)** Root, shoot, and total dry biomass (mg) of non-inoculated control and *P. thivervalensis* SC5 inoculated plants under normal and salt stress conditions; **(C)** Non-inoculated control and *P. thivervalensis* SC5 inoculated cucumber plants under salt stress conditions (100mM NaCl). NIC-0- non-inoculated control, normal conditions; SC5-0- *P. thivervalensis* SC5 inoculation, normal conditions; NIC-100- non-inoculated control, salt stress conditions (100mM NaCl); SC5-100- *P. thivervalensis* SC5 inoculation, salt stress conditions (100mM NaCl). RDW, root dry weight; SDW, shoot dry weight; and TDB, total dry biomass. Different letters adjacent to bars represent significant statistical differences (*p*<0.05).

The results obtained from the cucumber growth promotion assay conducted under greenhouse conditions showed that cucumber plants inoculated with *P. thivervalensis* SC5 presented a significant increased development when compared with non-inoculated plants, in both normal and salt stress conditions (0 and 100mmol/l NaCl, respectively; Figures 1A–C).

Compared to the non-inoculated control, the application of strain SC5 led to a significant increase in cucumber SL (8.4 vs. 11.5cm, 36.9%), root length (RL; 18.67 vs. 23.75cm, 27.2%), root dry weight (RDW; 77.30 vs. 102.32mg, 32.3%), shoot dry weight (SDW; 347.47 vs. 428.52mg, 23.3%), and total dry biomass (TDB; 424.80 vs. 530.83mg, 25%) in the absence of added salt (Figures 1A,B).

In the presence of salt there was a significant decrease in root and shoot growth (43% shoot, 22.3% RL) and in RDW (60.2%), SDW (34.6%), and TDB (39.3%) in non-inoculated cucumber plants (Figures 1A,B). Similar results were obtained with cucumber plants inoculated with *P. thivervalensis* SC5 under salt stress conditions, with decreases of 45.2, 20.35, 50.9, 23.3 and 28.6% in SL, RL, RDW, SDW, and TDB, respectively, when compared to SC5 inoculated plants under normal conditions. The effects of salt stress, especially those affecting biomass, were less pronounced in plants inoculated with *P. thivervalensis* SC5.

Compared to non-inoculated plants, the plants inoculated with *P. thivervalensis* SC5 showed a significant increase in both SL (4.80 vs. 6.25cm, 30.2%), RL (14.5 vs. 18.92cm, 30.5%), RDW (30.78 vs. 50.23mg, 63.2%), SDW (227.36 vs. 328.88mg, 44.7%), and TDB (258.14 vs. 379.12mg, 46.9%), under salt stress conditions (Figures 1A–C). Moreover, the results showed that SC5-inoculated salt-stressed plants had similar biomass values to non-inoculated non-stressed control plants (Figure 1B).

The results obtained here are in agreement with previous reports demonstrating the important role of ACC deaminase-producing bacteria (especially *Pseudomonas*) in mitigating the effects of salt stress in several plants (Cheng et al., 2007; Saravanakumar and Samiyappan, 2007; Ali et al., 2014; Orozco-Mosqueda et al., 2019), including cucumber (Gamalero et al., 2010).

### Detailed Analysis of *Pseudomonas thivervalensis* SC5 Genomic Properties

**Figure 2:**
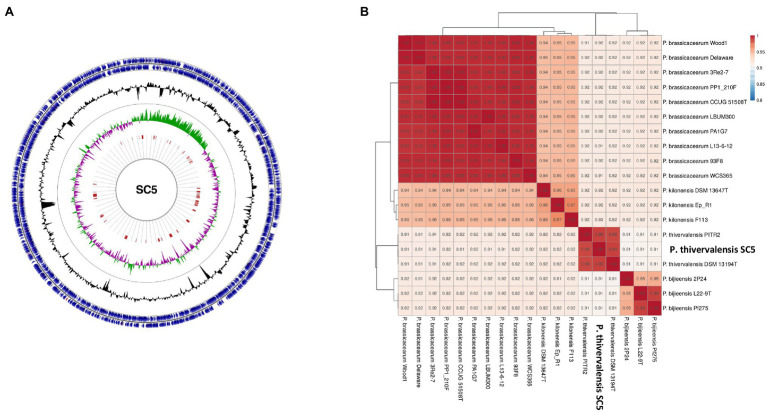
Representation of the *P. thivervalensis* SC5 genome. **(A)** Circular representation of the genome and its properties, including genes (blue), GC% (black), GC% skew [(+) green, and (−) pink] and genomic islands (red); **(B)** Phylogenomic analysis based on average nucleotide identity (ANI) values of selected *Pseudomonas* strains.

The genome of *P. thivervalensis* SC5 consists of a single circular chromosome of ~6.59Mbp and an average GC content of 61.2% (Figure 2A). Genome analysis predicts a total of 5,884 open reading frames, of which 5,816 correspond to putative protein coding sequences (CDS). A total of 58 tRNA, seven rRNA, three ncRNA, and one tmRNA genes were also found.

BlastKoala analysis resulted in the functional annotation of 3,380 genes from a total of 5,816 CDS (58.1%).

The genome of strain SC5 contains 33 genomic islands (GI; Figure 2A). Two of these GIs correspond to phage sequences that were found by PHAST analysis. A total of two complete phage sequence clusters were found within the SC5 genome.

CAZymes analysis identified 157 proteins belonging to the families of structurally related catalytic and carbohydrate-binding modules (or functional domains) of enzymes that degrade, modify, or create glycosidic bonds. A total of 42 proteins were predicted to belong to the Glycoside Hydrolase family, 38 to Glycosyl Transferases, 14 to Carbohydrate Binding Modules, 40 to Carbohydrate Esterases, 18 to Auxiliary Activities, and to 5 the Polysaccharide Lyases family.

The complete elements for *sec* and *tat*, type I (T1SS; Hemophore/metalloprotease transporter; Adhesin protein transporter; and AlgE-type Mannuronan C-5-Epimerase transporter), two type II (T2SS), a type III (T3SS), and two type VI (T6SS) secretion systems gene clusters were identified within the SC5 genome (Supplementary Table S1).

Genome alignment analysis revealed that the genome of *P. thivervalensis* SC5 is highly syntenic to the genomes of *P. thivervalensis* BS3779=DSM 13194^T^ and *P. thivervalensis* PITR2 (Supplementary Figure S2).

Strain SC5 was previously classified as *P. thivervalensis* based in its 16S rRNA gene sequence (Nascimento et al., 2019). To validate the previous taxonomic classification a genome-based analysis was conducted. The ANI analysis indicated that strain SC5 genome presents high identity to *P. thivervalensis* BS3779^T^ (ANI-98.69%) and *P. thivervalensis* PITR2 (ANI-98.62%) genomes (Figure 2B), supporting the identification of strain SC5 as *P. thivervalensis*. Moreover, strain SC5 as well as other *P. thivervalensis* strains presented increased genomic identity when compared to members of the *Pseudomonas brassicacearum*, *Pseudomonas kilonensis*, and *Pseudomonas bijeensis* species (Figure 2B). Interestingly, these clades contain well known PGPB also presenting pathogen antagonistic activities (e.g., *P. brassicacearum* WCS 365, *P. kilonensis* F113, and *P. brassicacearum* LBUM300).

### *Pseudomonas thivervalensis* SC5 Genomic Traits Involved in Soil Colonization, Plant-Growth Promotion, and Protection

#### Nitrogen, Sulfur, and Phosphorous Metabolism

*Pseudomonas thivervalensis* SC5 reduces nitrate to produce N (gas) and ammonia, indicating that the dissimilatory nitrate reduction and denitrification pathways are active in this strain. These results are consistent with the presence of the genes responsible for the dissimilatory nitrate reduction (*nas*) and denitrification (*nir, nar,* and *nor*) pathways, as well as several genes involved in nitrate, nitrite, and ammonia transport (three *amtB* genes) in the SC5 genome (Supplementary Table S2). Additionally, genes involved in urea and allophanate transport and degradation, namely the *ureABC* cluster responsible for urease production and, *atzF,* encoding the allophanate hydrolase, were also found (Supplementary Table S2).

Two nitronate monooxygenase genes (*npd*) involved in the oxidative denitrification of toxic nitronates and nitroalkanes to their corresponding aldehydes and nitrites; and three copies of the *azoR* gene, encoding azoreductase were found to be present in the genome (Supplementary Table S2), and may account for strain’s SC5 ability to degrade nitro-containing xenobiotics.

Strain SC5 possesses the complete set of genes responsible for the assimilatory sulfate reduction pathway (*cis* genes) and several genes involved in sulfate transport (*sulP, cysPUWA*). The taurine transport and taurine dioxygenase genes (*tauD*, three copies; Supplementary Table S3) involved in the catabolism of taurine; sulfone and sulfonate transport and degradation genes involved in the degradation of sulfones (*sfnG*) and alkanesulfonates (*ssuABCD*); and *dddP* and *dmoA* genes involved in the catabolism of dimethlysulfonioproprionate and dimethylsulfide were also found in the genome, suggesting an ability to modulate plant and soil sulfur metabolism.

The genome of strain SC5 contains the pyrroloquinoline quinone (*pqq*) operon and glucose dehydrogenase genes involved in the production of gluconate, a major organic acid involved in the solubilization of mineral phosphate and other compounds such as ZnO. In addition, genes encoding enzymes involved in organic phosphate solubilization, such as an extracellular phytase, several alkaline phosphatases, and some components of the phosphonate C-P lyase system were found in the genome of strain SC5 (Supplementary Table S4). The strain SC5 extracellular phytase (CE140_04710) presents some similarity with *Bacillus subtilis* extracellular 3-phytase involved in the catabolism of phytate a common plant metabolite found in plant tissues and soils, that serves as a storage source for phosphorus and inositol (Zeng et al., 2011). Two copies of the genes encoding the phosphate transport system (*pstABCS*) were also detected in the genome (Supplementary Table S4).

Overall, the data obtained indicates that *P. thivervalensis* SC5 participates in N, S, and P soil cycles through several enzymatic activities, thus, increasing the availability of several key nutrients that are indispensable for plant growth.

#### Siderophore Production and Iron Acquisition

*Pseudomonas thivervalensis* SC5 and its relatives, *P. thivervalensis* LMG 21626^T^ (=BS3779) and *P. thivervalensis* PITR2, synthesize siderophores. Recently, Matthijs et al. (2016) identified two siderophores produced by strain LMG 21626^T^, as pyoverdine (PYO_thi_) and histocorrugatin, and characterized the genomic regions responsible for their biosynthesis. The genomic regions containing the *pvd* and *hcsABCDEFGHIJKL* genes were detected in the chromosome of *P. thivervalensis* SC5 (Supplementary Table S5), suggesting the production of pyoverdine (PYO_thi_) and histocorrugatin by this strain. In addition to pyoverdine and histocorrugatin biosynthetic clusters, one other region containing a Non-Ribosomal Peptide Synthase (NRPS) and other siderophore biosynthesis related genes was detected in the strain SC5 chromosome (Supplementary Table S5). This genomic region (CE140_5180-5260) contained both isochorismate synthase (CE140_05240) and isochorismate pyruvate lyase (CE140_05235) gene homologs, which are involved in the production of SA, suggesting the production of a SA-containing siderophore. Moreover, the NRPS associated with this cluster (CE140_5180, CE140_5185) showed some homology (~43–46%) to NRPS’s from the coelibactin biosynthetic cluster of *Streptomyces coelicolor* A3(2). BLAST analysis revealed that this NRPS cluster is somewhat rare, being only detected in 20 other sequenced *Pseudomonas* strains (mostly *synxantha, simiae, libanensis*, and *azotoformans*).

Iron and iron complex (including siderophore-iron complex) transport genes are extremely abundant in the genome sequence of strain SC5 (Supplementary Table S5), suggesting that iron acquisition and metabolism plays an important role in strain SC5 physiology and ecology.

#### Osmotic Stress Resistance

Strain SC5 was able to grow in the presence of high salt concentrations, which is consistent with the presence of multiple genes involved in the biosynthesis, metabolism and transport of osmoprotectants in its genome (Supplementary Table S6). In this regard, strain SC5 possesses the genes for the biosynthesis of compatible solutes such as proline (*proABC*), glutamate (*gltBD*), glutamine (seven *glnA* genes), carnitine (*bodG, lcdH*), N-acetylglutaminylglutamine amide (NAGGN; *ngg*), glycine-betaine (*betABC*), glycogen (*glg*), trehalose (two *treS* genes, *treYZ*), ectoine, and hydroxyectoine (*doeABCD*), as well as several of the transporters of these compounds (*proP, proXWV, betT, opuABCD, thuEFGK,* and *ehuABCD;* Supplementary Table S6). In addition, several genes involved in the transport of sodium and chloride (*nhaAB*, *yfbK*, *glnT,* and *putP*) were detected in the genome.

The SC5 genome also contains several genes involved in the biosynthesis, metabolism, and transport of polyamines and 4-aminobutyrate (GABA; Supplementary Table S7), which also play a role in salt stress resistance (Schiller et al., 2000). The strain SC5 chromosome contains the *speA, aguA, aguB*, and *speC* genes involved in putrescine biosynthesis; the *hss*, *cansD*, and *nspC* genes involved in homospermidine, carboxynorspermidine, and spermidine biosynthesis; as well as several genes involved in polyamine degradation (*puuABCD*, *spuC*, and *prr*) and conversion to GABA.

The results obtained indicate that *P. thivervalensis* SC5 can acquire, synthesize, accumulate, and metabolize wide range of osmolytes and polyamines that are known to play a role in osmotic stress tolerance, as well as in plant colonization and plant growth promotion. For example, the induction of osmotic stress and the presence of compatible solutes increased the plant colonization and biocontrol activities of *P. fluorescens* EPS62e (Bonaterra et al., 2007). The production of trehalose by the PGPB *Pseudomonas* sp. UW4, played an important role in the protection of tomato plants against salt stress (Orozco-Mosqueda et al., 2019). The biosynthesis of polyamines plays a role in plant growth promotion activities of plant-associated bacteria (Chen et al., 2017).

#### Motility, Chemotaxis, and Attachment to Plant Tissues

*Pseudomonas thivervalensis* SC5 chromosomal DNA contains the genes involved in the synthesis of flagella (*flg, fli*) and pilus (*pil, cpa*) systems involved in bacterial motility (Supplementary Table S8). The genome also harbors 38 genes encoding methyl-accepting chemotaxis proteins as well as multiple copies of chemotaxis related genes *cheA, cheB, cheD, cheV, cheY, cheR, motA,* and *motB* (Supplementary Table S8).

The SC5 genome harbors several genes involved in bacterial attachment to plant tissues, including the type IV pilus and fimbriae biosynthesis genes, the tight adherence (Tad) export apparatus and Flp (fimbrial low molecular-weight protein) pili, the *bcs* cellulose production operon, the *alg* operon responsible for alginate production, and several other gene clusters potentially involved in exopolysaccharide formation (Supplementary Table S8).

#### Resistance to Oxidative Stress

*Pseudomonas thivervalensis* SC5 harbors an increased number of genes involved in the response to oxidative stress, including four *katE* genes encoding catalase; *katG*, encoding a catalase-peroxidase; two *sod* genes that encode Fe-Mn family superoxide dismutases; two *ahpC* genes that encode an alkyl hydroperoxide reductase; a peroxiredoxin Q/BCP *bcp* gene*; tpx,* encoding an atypical 2-Cys peroxiredoxin; a cytochrome c peroxidase gene; three *gpx* genes encoding glutathione peroxidase; four non-heme chloroperoxidases *(cpo)*; three *osmC* genes that encode a lipoyl-dependent peroxiredoxin; a dye-decolorizing peroxidase gene homolog, and a superoxide oxidase, *cybB,* gene. A gene cluster involved in the production of an aryl polyene, possibly involved in the protection against reactive oxygen species (ROS; Cimermancic et al., 2014), was also detected in the genome of *P. thivervalensis* SC5 (Supplementary Table S9). This data indicates that *P. thivervalensis* SC5 possess a significant ability to detoxify several ROS, which may impact several aspects of plant growth as well as plant-microbe interactions. For example, plants produce increased levels of ROS under stressful conditions (e.g., salt stress) that impact plant growth (Huang et al., 2019). The plant defense response against bacteria is mostly mediated by ROS (Bolwell and Wojtaszek, 1997). Ultimately, beneficial bacteria able to overcome the inhibitory effects of ROS are better able to colonize and protect plant hosts against stress (Kim et al., 2000; Alquéres et al., 2013; Tiepo et al., 2020).

#### Metabolism of Carbohydrates, Organic Acids, and Amino Acids

Genomic analysis revealed the presence of several carbohydrate (sugars, sugar alcohols, and sugar acids) catabolism and transport genes in strain SC5 chromosomal DNA (Supplementary Table S10). These included genes involved in the catabolism of sugars such as glucose (through gluconate), fructose, ribose, xylose, galactose, mannose, sucrose, maltose, trehalose, and beta-glucosides; sugar alcohols such as glycerol, mannitol, galactictol/sorbitol, and inositol; and sugar acids such as glycolate, D-gluconate, L-gulonate, D-fructuronate, D-mannonate, D-lactate, galactarate, glycerate, D-glucarate, L-talarate, and D-galactonate degradation. The SC5 genome also contains the genes involved in the TCA cycle (citrate, fumarate, succinate, aconitate, isocitrate, and malate metabolism), the glyoxylate bypass (glyoxylate catabolism), and the metabolism of several organic acids such as acetate, oxaloacetate, formate, malonate, tartrate, propionate, and butyrate (Supplementary Table S11).

Strain SC5 also contains several genes involved in the transport and metabolism of amino acids and peptides, as well as opines (Supplementary Table S12). Opines are compounds synthesized from the condensation of amino acids with ketoacids or sugars, and have been linked to the colonization abilities of rhizobia and *Agrobacterium* (Murphy et al., 1995; Dessaux et al., 1998). The genome of strain SC5 contains the *mocC* gene involved in the catabolism of rhizopine (Galbraith et al., 1998), several opine oxidase gene homologs (*ooxAB*) involved in octopine degradation, as well as the inositol/myo-inositol transport system and homologs of the octopine transport system, indicating the strain SC5 ability to catabolize opines and modulate its effects on plant-microbe interactions.

#### Phenolics and Lignin Metabolism

The *P. thivervalensis* SC5 genome contains the *ech*, *vdh*, and *fcs* gene cluster that encodes the enzymes enoyl-CoA hydratase/aldolase, vanillin dehydrogenase, and feruloyl-CoA synthetase, involved in the catabolism of hydroxycinnamic acids such as ferulic acid (conversion to vannilate), p-coumaric acid (conversion to 4-hydroxybenzoate) and caffeic acid (conversion to protocatechuate). A *mhpA* gene homolog encoding the 3-(3-hydroxyphenyl)propanoate hydroxylase involved in 3-hydroxycinnamic acid (m-coumaric acid) catabolism, and the *hpa* operon encoding a 4-hydroxyphenylacetate 3-monooxygenase system involved in p-coumaric acid oxidation, are also present in the chromosome of strain SC5. Moreover, the vannilate catabolism genes, *vanAB*, responsible for the conversion of vannilate to protocatechuate; the *pobA* gene responsible to the conversion of 4-hydroxybenzoate to protocatechuate; and the *pca* operon containing the genes responsible for the conversion of protocatechuate to acetyl-CoA (*via* 3-oxoadipate) were also detected (Supplementary Table S13), consistent with the presence of a complete degradation pathway for hydroxycinnamic acids in strain SC5.

The ability to use hydroxycinnamic acids as carbon sources may play a role in *P. thivervalensis* SC5 rhizospheric colonization activities, as well as in soil and plant health maintenance by decreasing the long-term accumulation of these toxic allelochemical compounds (Turner and Rice, 1975; Batish et al., 2008; Singh et al., 2014; Ferro et al., 2015).

Two NADPH-dependent curcumin reductase genes (*curA*) were also detected in the SC5 genome (Supplementary Table S13) and may be involved in strain SC5 ability to metabolize the plant polyphenolic and antibacterial compound curcumin.

A gene encoding a Dyp type peroxidase enzyme (CE140_09525) with 84.5% identity to the *P. fluorescens* Pf-5 DyP1B enzyme, involved in the oxidation of lignin substrates (Rahman Pour and Bugg, 2015), was found in the genome of *P. thivervalensis* SC5, suggesting that this strain has the ability to degrade lignin.

Genomic analysis also revealed the presence a polyphenol oxidase/laccase encoding gene, responsible for the oxidation of several phenolics; *calA* and *calB* genes encoding the coniferyl-alcohol dehydrogenase and coniferyl-aldehyde dehydrogenase enzymes, respectively, involved in the catabolism of the monolignol coniferyl-alcohol and the respective coniferyl aldehyde (Supplementary Table S13); a 2,4'-dihydroxyacetophenone dioxygenase responsible for the degradation of 2,4'-dihydroxyacetophenone to 4-hydroxybenzoate; and the catechol 2,3-dioxygenase enzyme responsible for the catabolism of catechol. Coniferyl-alcohols and hydroxycinnamic acids are components of plant lignin, and 2,4'-dihydroxyacetophenone and catechol are known breakdown products of lignin. Overall, the acquired data suggests that strain SC5 may modulate plant lignin levels, which are an important determinant of plant growth, stress resistance, and defense (Liu et al., 2018).

#### Metabolism of Flavonoids

SC5 genomic analysis led to the identification of a cluster (CE140_08155-08295) containing several genes that present a high level of identity to the naringenin degradation genes of *Herbaspirillum seropedicae* SmR1 (Marin et al., 2013). In addition, three quercetin 2,3-dioxygenase genes, involved in the catabolism of the flavonoid quercetin were also detected in the genome of *P. thivervalensis* SC5 (Supplementary Table S13).

Flavonoid degradation is a trait described in several *Pseudomonas* species (Rao and Cooper, 1994; Pillai and Swarup, 2002) that may play an important role in the modulation of soil and plant flavonoid concentrations. The degradation of flavonoids leads to the production of p-coumaric acid, caffeic acid, phenylacetic acid (PAA), p-hydroxybenzoate, and protocatechuate (B-ring products); and phloroglucinol, phloroglucinol carboxylate, resorcinol, and oxaloacetate (A-ring products; Rao and Cooper, 1994; Pillai and Swarup, 2002), which are then channeled to the TCA cycle. Most of the genes involved in these pathways were found in the SC5 genome, indicating the ability to completely use several flavonoids as carbon sources, a trait likely associated with the colonization activities of the bacterium.

#### ACC and Ethylene Modulation Genes

The *acdS* gene (CE140_07955) encoding ACC deaminase, as well as the *acdR* gene (CE140_07960) that encodes a leucine-responsive protein known to regulate *acdS* gene expression (Li and Glick, 2001), were found in the genome of *P. thivervalensis* SC5 (Supplementary Table S14). The AcdS and AcdR proteins present a high identity (97.6 and 86.4%, respectively) to those of the efficient ACC deaminase-producing PGPB, *Pseudomonas* sp. UW4. Moreover, analysis of the *P. thivervalensis* SC5 *acdS* gene and its nearby regulatory regions indicated that this gene is stably present in the SC5 chromosome (with no transposase or mobile genetic elements in its vicinity), indicating a long-term acquisition and evolution of the *acdS* gene in strain SC5. The *acdS* gene is known to be positively selected in most legume-rhizobia symbiosis (Nascimento et al., 2018b); hence, the presence of a stable *acdS* gene in *P. thivervalensis* SC5 suggests that this strain has evolved a long term mutualistic interaction with plant hosts, where the expression of ACC deaminase plays a valuable and positive role.

In addition to *acdS*, strain SC5 also contains a *tlpQ* gene homolog (CE140_07340), involved in the chemotactic response to ethylene, which acts as an attractant to several plant-associated *Pseudomonas* strains (Kim et al., 2007); and several genes involved in polyamine (putrescine, spermidine) production and transport (Supplementary Table S14). Bacterial spermidine inhibits plant ethylene biosynthesis, leading to an increase in plant growth promotion (Xie et al., 2014).

#### Auxin Biosynthesis

A tryptophan 2-monooxygenase gene (*iaaM*) homolog, possibly involved in the biosynthesis of indole-3-acetamide (IAM), and several amidase genes (*amiE, yafV*) that may convert IAM to IAA, were found in the genome of *P. thivervalensis* SC5 (Supplementary Table S14), thus suggesting that strain SC5 produces IAA trough the IAM pathway (Duca and Glick, 2020). The production of IAA trough the IAM pathway has also been described in other beneficial *Pseudomonas* strains, such as *Pseudomonas chlororaphis* O6 (Dimkpa et al., 2012).

Curiously, two *feaB* genes encoding the phenylacetaldehyde dehydrogenase enzyme involved in phenylacetate (PAA) production were identified in the genome of strain SC5 (Supplementary Table S14), thus suggesting its ability to synthesize PAA. PAA is also considered an auxin with significant impact in plant growth (Cook, 2019). Moreover, PAA production by beneficial plant-associated bacteria also acts as an inducer of the plant defense response, leading to increased plant resistance against fungal pathogens (Sumayo et al., 2013; Akram et al., 2016).

#### Cytokinins Biosynthesis and Metabolism

The genome of strain SC5 contains several genetic elements involved in the production and transformation of cytokinins (CKs; Supplementary Table S14), such as the *miaA, miaB*, and *miaE* genes involved in the production of N6-(Δ2-isopentenyl)adenosine, 2-methylthio-N6-(dimethylallyl)adenosine, and 2-methylthio-cis-ribozeatin, respectively. The SC5 genome also contain several genes encoding RNAses, which can be involved in the dissociation of tRNA-associated CKs. Additionally, two *log* genes, which encode the cytokinin riboside 5'-monophosphate phosphoribohydrolase responsible for the conversion of CK-nucleotides to free-base forms (Samanovic et al., 2015); and the xanthine dehydrogenase genes, *xdhABC*, responsible for the biotransformation of CKs (Taylor et al., 2006), were also found in the genome sequence of *P. thivervalensis* SC5 (Supplementary Table S14).

The abundance of genetic elements involved in the production and transformation of CKs suggests that *P. thivervalensis* SC5 is able to synthesize CKs, a trait also found in other *Pseudomonas* strains (de Salamone et al., 2001; Karnwal and Kaushik, 2011; Großkinsky et al., 2016). Moreover, *P. fluorescens* G20-18 CK production abilities also play an important role in the biocontrol of *P. syringae* infection in *Arabidopsis* (Großkinsky et al., 2016); hence, the production of CKs by *P. thivervalensis* SC5 may impact not only its plant growth promotion activities but also its biocontrol activities.

#### Salicylic Acid Biosynthesis

Several genes involved in the production of SA, namely, one isochorismate synthase (*pchA*), which converts chorismate into isochorismate (the building block for SA biosynthesis) and two isochorismate-pyruvate lyase gene homologs (*pchB*), which are responsible for the transformation of isochorismate to SA, were identified in the SC5 chromosomal DNA (Supplementary Table S14). The *pchA* and one copy of the *pchB* gene were found in an operon responsible for siderophore biosynthesis (described above), suggesting a role in the synthesis of a SA-containing siderophore. One additional copy of *pchB (pchB2)* was found in a genomic region not related to siderophore production; *pchB2* (CE140_03015) is clustered with a gene encoding an isochorismatase-like protein. These results suggest the production of SA by *P. thivervalensis* SC5, a trait that has also been described in other *Pseudomonas* spp. (Bakker et al., 2014).

Salicylic acid is one of the main plant hormones involved in plant defense responses, and, consequently, has important functions in regulating plant-microbe interactions (Lebeis et al., 2015). Moreover, SA also plays an important role in plant growth and abiotic stress resistance (Rivas-San Vicente and Plasencia, 2011; Khan et al., 2015). The production of SA by *P. thivervalensis* SC5 may play an important role in its ability to interact with plant hosts, to promote plant growth as well as in its biocontrol activities against pathogens.

#### Phytopathogen Antagonistic Activities

ANTISMASH analysis revealed the presence of several gene clusters involved in the biosynthesis of antagonistic secondary metabolites in the genome of strain SC5. These included a 2,4-diacetylphloroglucinol (DAPG) biosynthesis cluster constituted by the *phlHGF* and *phlACBDE* genes; a cluster involved in hydrogen cyanide (HCN) production (*hcnABC*); a gene cluster involved in the biosynthesis of a fragin homolog; three gene clusters involved in bacteriocin production, and one cluster involved in the production of a lantipeptide (Supplementary Table S15).

Strain SC5 DAPG biosynthesis cluster (*phlACBDE* and *phlHGF*) presents high identity to the functional DAPG biosynthesis gene cluster of *P. thivervalensis* PITR2 (~99% identity; Keel et al., 1996; Molina et al., 2003), and other studied biocontrol *Pseudomonas* strains such as *P. kilonensis* F113 (~93% identity; Redondo-Nieto et al., 2013; Vacheron et al., 2018), *P. brassicacearum* BIM B-446 (~93% identity; Mandryk-Litvinkovich et al., 2017), *P. brassicacearum* 3Re2-7 (~93% identity; Nelkner et al., 2019), *P. brassicacearum* LBUM300 (~93% identity; Lanteigne et al., 2012; Novinscak et al., 2016), among others. Moreover, BLAST analysis revealed that the *hcnABC* gene cluster of *P. thivervalensis* SC5 also presented high identity to the functional *hcnABC* genes of several HCN-producing *Pseudomonas*, including *P. thivervalensis* PITR2 (~99% identity; Ramette et al., 2003), *P. brassicacearum* 3Re2-7 (~92% identity; Nelkner et al., 2019) and *P. brassicacearum* LBUM300 (~92% identity; Lanteigne et al., 2012; Novinscak et al., 2016).

Several genes involved in acyl homoserine lactone catabolism (*pvdQ*, *quiP*) and homoserine lactone efflux were detected in the genome of strain SC5 (Supplementary Table S15), suggesting the ability to modulate quorum-sensing signals, a trait of relevant importance for biocontrol activities (Alymanesh et al., 2016).

**Figure 3:**
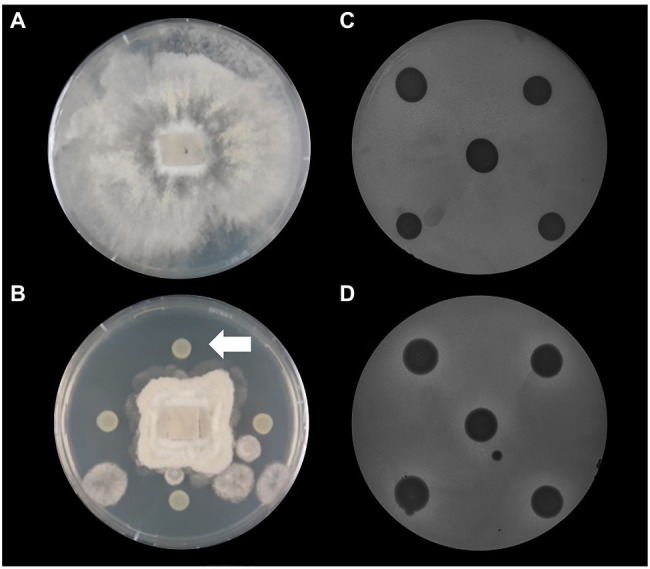
Antagonistic activities against phytopathogens. **(A)** *Botrytis cinerea* control; **(B)** *B. cinerea*+*P. thivervalensis* SC5; **(C)** *Pseudomonas syringae* DC3000 (incorporated in agar)+*Pseudomonas* sp. MS8 (negative control); **(D)** *P. syringae* DC3000 (incorporated in agar)+*P. thivervalensis* SC5. The arrow in panel **(B)** points to a *P. thivervalensis* SC5 colony. The inhibition of fungal growth and the presence of halos (*P. syringae* DC3000 assay) indicate the antagonistic activities of strain SC5.

Based on the increased prevalence of genes involved in the biosynthesis of antagonistic secondary compounds it was decided to test *P. thivervalensis* SC5 for its ability to antagonize the phytopathogens *B. cinerea* (fungal) and *P. syringae* DC3000 (bacterial). These assays revealed that *P. thivervalensis* SC5 inhibited the growth of both pathogens, confirming the active production of pathogen antagonistic traits (Figure 3). The obtained results indicate that strain SC5 presents the ability to act as a biocontrol agent, a trait also observed in its close relative, *P. thivervalensis* PITR2, a bacterium isolated from a disease suppressive soil known to actively produce the antifungal metabolites DAPG and HCN [73].

## Conclusion

**Figure 4:**
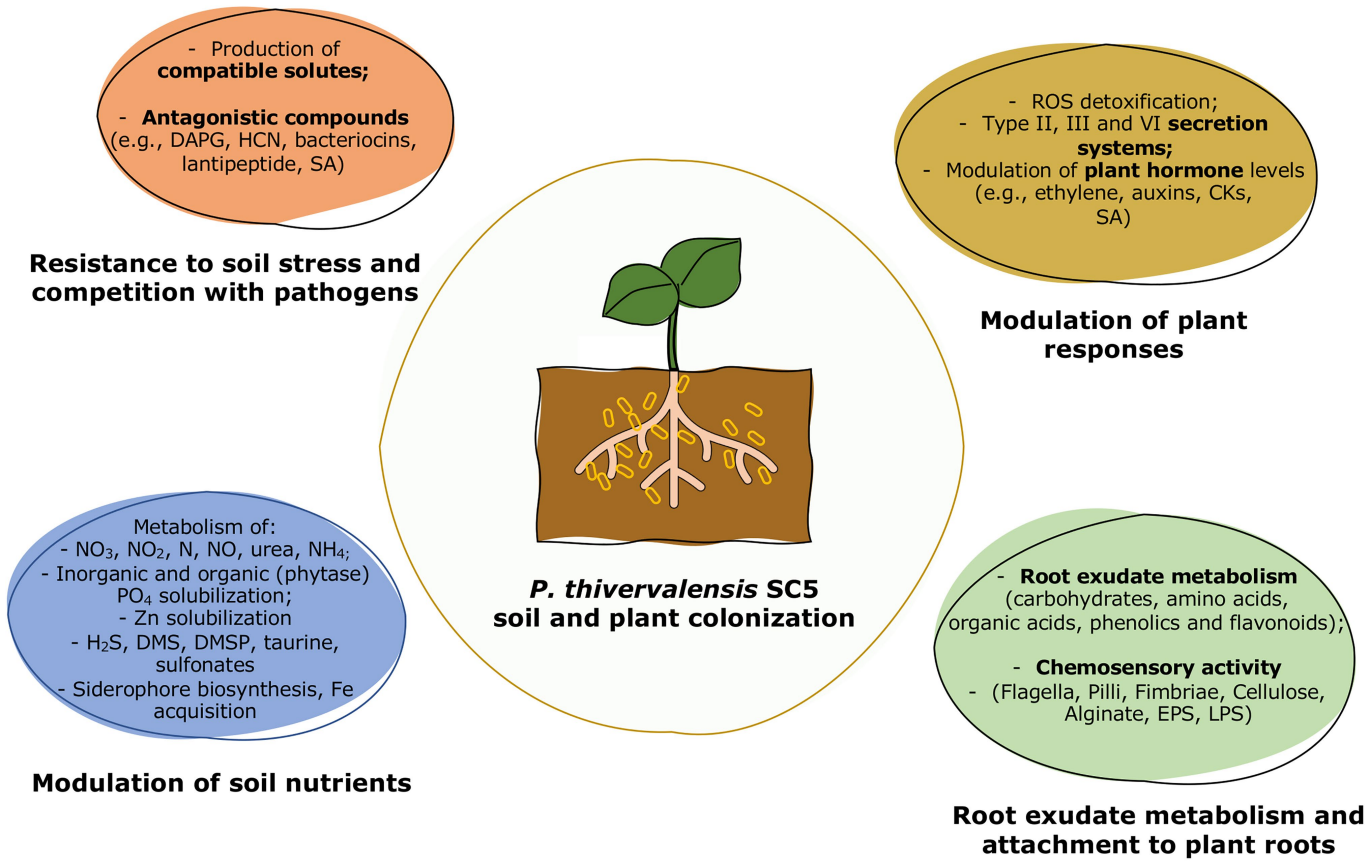
Overview of the *P. thivervalensis* SC5 soil and plant colonization activities.

*Pseudomonas thivervalensis* SC5 acts as a versatile and efficient PGPB, presenting both plant growth promoting properties as well as antagonistic activities against phytopathogens. Although, this strain was originally isolated based on its ACC deaminase activity, a thorough genomic analysis revealed the presence of several other elements involved in plant colonization and growth promotion. Thus, the success of *P. thivervalensis* SC5 as a PGPB results from a web of complex mechanisms and actions that modulate several aspects of plant growth, resistance, and plant-microbe interactions (Figure 4; Table 2).

**Table 2:** Features encoded in the *P. thivervalensis* SC5 genome and their roles in plant growth promotion, plant-microbe interactions, and phytopathogen antagonism. Detailed analysis of genes can be found in Supplementary Tables S1–S15.

Genome encoded features	Major roles
Metabolism of: NO_3_, NO_2_, N, NO, urea, and NH_4_	• Modulation of soil and plant N levels
	• Induction of plant defense response and systemic resistance
Inorganic and organic (phytase) PO4 solubilization;	• Modulation of soil and plant P levels
Zn solubilization	• Availability of important co-factors (Zn)
Metabolism of: H2S, DMS, DMSP, taurine, and sulfonates	• Modulation of soil and plant S levels
Siderophore biosynthesis (Pyoverdine, histocorrugatin, and SA-containing siderophore); Siderophore/Iron acquisition	• Modulation of soil and plant Fe levels
	• Antagonism to phytopathogens (biocontrol)
Chemosensory activity, Flagella, Pilli, Fimbriae, Cellulose, Alginate, EPS, and LPS	• Motility, chemotaxis and attachment to plant tissues
	• Colonization activities
	• Induction of plant defense response and systemic resistance
Biosynthesis and metabolism of: proline, glutamate, glutamine, carnitine, NAGGN, glycine-betaine, glycogen, trehalose, ectoine, hydroxyectoine, putrescine, homospermidine, carboxynorspermidine spermidine, and GABA	• Resistance to osmotic stress
	• Plant protection against osmotic stress
	• Modulation of ethylene levels (by spermidine)
Catalases, SODs, peroxiredoxins, and aryl polyene	• Resistance to ROS
	• Resistance to plant defenses (endophytic colonization)
	• Plant protection against stress (e.g., osmotic, drought)
Metabolism of fructose, ribose, xylose, galactose, mannose, sucrose, maltose, trehalose, beta-glucosides; glycerol, mannitol, galactictol/sorbitol,inositol; glycolate, D-gluconate, L-gulonate, D-fructuronate, D-mannonate, D-lactate, galactarate, glycerate, D-glucarate, L-talarate, and D-galactonate; citrate, fumarate, succinate, aconitate, isocitrate, malate, acetate, oxaloacetate, formate, malonate, tartrate, propionate, butyrate; amino acids, and peptides, opines.	• Soil nutrient recycling
	• Catabolism of root exudates
	• Plant colonization
	• Resistance to plant defenses (peptides)
Metabolism of ferulic acid, p- and m-coumaric acid, caffeic acid, vannilate, 4-hydroxybenzoate; curcumin, lignin, coniferyl-alcohol, coniferyl-aldehyde; naringenin, quercetin, and other flavonoids	• Soil nutrient recycling
	Catabolism of root exudates (phenolics and flavonoids)
	• Plant colonization
	• Resistance to plant defenses
	• Induction of plant defense response and systemic resistance
ACC deaminase, IAA, PAA, CKs, and SA biosynthesis	• Plant colonization
	• Plant growth promotion
	• Resistance to plant defenses
	• Plant protection against stress
	• Antagonism to phytopathogens (biocontrol; SA)
Biosynthesis of DAPG, HCN, fragin homolog, bacteriocins, and lantipeptide; Acyl homoserine lactone catabolism	• Antagonism to phytopathogens (biocontrol)
Type II, Type III and Type VI secretion systems	• Colonization activities
	• Induction of plant defense response and systemic resistance
	• Antagonism to phytopathogens (biocontrol)

Ultimately, understanding the functions of successful PGPB, such as *P. thivervalensis* SC5 is of importance for the selection and development of next generation inoculants to be applied in a wide range of agricultural and biotechnological applications, and to gain more knowledge regarding beneficial plant microbe-interactions.

## Data Availability Statement

The datasets presented in this study can be found in online repositories. The names of the repository/repositories and accession number(s) can be found at: https://www.ncbi.nlm.nih.gov/genbank/, CP022201.1.

## Author Contributions

FN performed the plant experiments, bacterial characterization assays, genomic analysis, and wrote the manuscript. PU performed the plant experiments (greenhouse assays) and participated in manuscript writing. BG, AG, and MR were responsible for advising, writing, and revising this work. All authors contributed to the article and approved the submitted version.

## Conflict of Interest

The authors declare that the research was conducted in the absence of any commercial or financial relationships that could be construed as a potential conflict of interest.

## Publisher’s Note

All claims expressed in this article are solely those of the authors and do not necessarily represent those of their affiliated organizations, or those of the publisher, the editors and the reviewers. Any product that may be evaluated in this article, or claim that may be made by its manufacturer, is not guaranteed or endorsed by the publisher.

## Footnotes

^1^ https://www.ncbi.nlm.nih.gov/

## Abbreviations

PGPB: Plant-growth-promoting bacteria
ACC: 1-aminocyclopropane-1-carboxylic acid
IAA: Indole-3-acetic acid
SA: Salicylic acid
BA: Benzoic acid
ABA: Abscisic acid
JA: Jasmonic acid
GA: Gibberellic acid (GA)
PAA: Phenylacetic acid
CKs: Cytokinins
GABA: 4-aminobutyrate
SL: Shoot length
RL: Root length
RDW: Root dry weight
SDW: Shoot dry weight
TDB: Total dry biomass
CDS: Coding sequences
GI: Genomic islands
TnSS: Type n secretion system
ANI: Average nucleotide identity
ROS: Reactive oxygen species
SODs: Superoxide dismutases
DMS: Dimethylsulfide
DMSP: Dimethylsulfoniopropionate
DAPG: 2,4-diacetylphloroglucinol
HCN: Hydrogen cyanide
EPS: Exopolysaccharide
LPS: Lipopolysaccharide
NAGGN: N-acetylglutaminylglutamine amide

## References

[ref1] Akram W. Anjum T. Ali B. (2016). Phenylacetic acid is isr determinant produced by *Bacillus fortis* iags162, which involves extensive re-modulation in metabolomics of tomato to protect against *Fusarium* wilt. Front. Plant Sci. 7:498. doi: 10.3389/fpls.2016.00498 PMC4835451 27148321

[ref2] Ali S. Charles T. C. Glick B. R. (2014). Amelioration of high salinity stress damage by plant growth-promoting bacterial endophytes that contain ACC deaminase. Plant Physiol. Biochem. 80, 160–167. doi: 10.1016/j.plaphy.2014.04.003, PMID: 24769617

[ref3] Alquéres S. Meneses C. Rouws L. Rothballer M. Baldani I. Schmid M.. (2013). The bacterial superoxide dismutase and glutathione reductase are crucial for endophytic colonization of rice roots by *Gluconacetobacter diazotrophicus* PAL5. Mol. Plant Microbe Interact. 26, 937–945. doi: 10.1094/MPMI-12-12-0286-R 23634840

[ref4] Alymanesh M. R. Taheri P. Tarighi S. (2016). *Pseudomonas* as a frequent and important quorum quenching bacterium with biocontrol capability against many phytopathogens. Biocontrol Sci. Tech. 26, 1719–1735. doi: 10.1080/09583157.2016.1239065

[ref5] Angiuoli S. V. Gussman A. Klimke W. Cochrane G. Field D. Garrity G. M.. (2008). Toward an online repository of standard operating procedures (SOPs) for (meta)genomic annotation. OMICS 12, 137–141. doi: 10.1089/omi.2008.0017, PMID: 18416670 PMC3196215

[ref6] Bakker P. A. H. M. Ran L. Mercado-Blanco J. (2014). Rhizobacterial salicylate production provokes headaches! Plant Soil 382, 1–16. doi: 10.1007/s11104-014-2102-0

[ref7] Batish D. R. Singh H. P. Kaur S. Kohli R. K. Yadav S. S. (2008). Caffeic acid affects early growth, and morphogenetic response of hypocotyl cuttings of mung bean (*Phaseolus aureus*). J. Plant Physiol. 165, 297–305. doi: 10.1016/j.jplph.2007.05.003, PMID: 17643552

[ref8] Belimov A. A. Dodd I. C. Hontzeas N. Theobald J. C. Safronova V. I. Davies W. J. (2009). Rhizosphere bacteria containing 1-aminocyclopropane-1-carboxylate deaminase increase yield of plants grown in drying soil via both local and systemic hormone signalling. New Phytol. 181, 413–423. doi: 10.1111/j.1469-8137.2008.02657.x, PMID: 19121036

[ref9] Bolwell G. P. Wojtaszek P. (1997). Mechanisms for the generation of reactive oxygen species in plant defence - A broad perspective. Physiol. Mol. Plant Pathol. 51, 347–366. doi: 10.1006/pmpp.1997.0129

[ref10] Bonaterra A. Cabrefiga J. Camps J. Montesinos E. (2007). Increasing survival and efficacy of a bacterial biocontrol agent of fire blight of rosaceous plants by means of osmoadaptation. FEMS Microbiol. Ecol. 61, 185–195. doi: 10.1111/j.1574-6941.2007.00313.x, PMID: 17391328

[ref11] Broekaert W. F. Delauré S. L. De Bolle M. F. C. Cammue B. P. A. (2006). The role of ethylene in host-pathogen interactions. Annu. Rev. Phytopathol. 44, 393–416. doi: 10.1146/annurev.phyto.44.070505.143440, PMID: 16602950

[ref12] Buxton R. (2011). Nitrate and Nitrite Reduction Test Protocols. Available at: https://www.asmscience.org/content/education/protocol/protocol.3660 (Accessed April 20, 2021).

[ref13] Chen L. Liu Y. Wu G. Zhang N. Shen Q. Zhang R. (2017). Beneficial rhizobacterium *bacillus amyloliquefaciens* SQR9 induces plant salt tolerance through spermidine production. Mol. Plant-Microbe Interact. 30, 423–432. doi: 10.1094/MPMI-02-17-0027-R, PMID: 28291380

[ref14] Cheng Z. Park E. Glick B. R. (2007). 1-aminocyclopropane-1-carboxylate deaminase from *Pseudomonas putida* UW4 facilitates the growth of canola in the presence of salt. Can. J. Microbiol. 53, 912–918. doi: 10.1139/W07-050, PMID: 17898846

[ref15] Cimermancic P. Medema M. H. Claesen J. Kurita K. Wieland Brown L. C. Mavrommatis K.. (2014). Insights into secondary metabolism from a global analysis of prokaryotic biosynthetic gene clusters. Cell 158, 412–421. doi: 10.1016/j.cell.2014.06.034, PMID: 25036635 PMC4123684

[ref16] Cook S. D. (2019). An historical review of phenylacetic acid. Plant Cell Physiol. 60, 243–254. doi: 10.1093/pcp/pcz004, PMID: 30649529

[ref17] Croes S. Weyens N. Janssen J. Vercampt H. Colpaert J. V. Carleer R.. (2013). Bacterial communities associated with *Brassica napus* L. grown on trace element-contaminated and non-contaminated fields: a genotypic and phenotypic comparison. Microb. Biotechnol. 6, 371–384. doi: 10.1111/1751-7915.12057, PMID: 23594409 PMC3917472

[ref18] Darling A. C. E. Mau B. Blattner F. R. Perna N. T. (2004). Mauve: multiple alignment of conserved genomic sequence with rearrangements. Genome Res. 14, 1394–1403. doi: 10.1101/gr.2289704, PMID: 15231754 PMC442156

[ref19] de Freitas J. R. Banerjee M. R. Germida J. J. (1997). Phosphate-solubilizing rhizobacteria enhance the growth and yield but not phosphorus uptake of canola (Brassica napus L.). Biol. Fertil. Soil 24, 358–364. doi: 10.1007/s003740050258

[ref20] de Salamone I. E. G. Hynes R. K. Nelson L. M. (2001). Cytokinin production by plant growth promoting rhizobacteria and selected mutants. Can. J. Microbiol. 47, 404–411. doi: 10.1139/w01-029 11400730

[ref21] de Souza R. Ambrosini A. Passaglia L. M. P. (2015). Plant growth-promoting bacteria as inoculants in agricultural soils. Genet. Mol. Biol. 38, 401–419. doi: 10.1590/S1415-475738420150053, PMID: 26537605 PMC4763327

[ref22] Dessaux Y. Petit A. Farrand S. K. Murphy P. J. (1998). “Opines and opine-like molecules involved in plant-rhizobiaceae interactions,” in The Rhizobiaceae. eds. Spaink H. P. Kondorosi A. Hooykaas P. J. J. (Netherlands: Springer), 173–197.

[ref23] Dimkpa C. O. Zeng J. McLean J. E. Britt D. W. Zhan J. Anderson A. J. (2012). Production of indole-3-acetic acid via the indole-3-acetamide pathway in the plant-beneficial bacterium *Pseudomonas chlororaphis* O6 is inhibited by ZnO nanoparticles but enhanced by CuO nanoparticles. Appl. Environ. Microbiol. 78, 1404–1410. doi: 10.1128/AEM.07424-11, PMID: 22210218 PMC3294495

[ref24] Dubois M. Van den Broeck L. Inzé D. (2018). The pivotal role of ethylene in plant growth. Trends Plant Sci. 23, 311–323. doi: 10.1016/j.tplants.2018.01.003 29428350 PMC5890734

[ref25] Duca D. R. Glick B. R. (2020). Indole-3-acetic acid biosynthesis and its regulation in plant-associated bacteria. Appl. Microbiol. Biotechnol. 104, 8607–8619. doi: 10.1007/s00253-020-10869-5, PMID: 32875364

[ref26] Ferro A. P. Marchiosi R. Siqueira-soares R. D. C. Bonini E. A. (2015). Effects of cinnamic and ferulic acids on growth and lignification of maize roots. J. Allelochem. Interact. 2015, 29–38.

[ref27] Galbraith M. P. Feng S. F. Borneman J. Triplett E. W. De Bruijn F. J. Rossbach S. (1998). A functional myo-inositol catabolism pathway is essential for rhizopine utilization by *Sinorhizobium meliloti*. Microbiology 144, 2915–2924. doi: 10.1099/00221287-144-10-2915, PMID: 9802033

[ref28] Gamalero E. Berta G. Massa N. Glick B. R. Lingua G. (2010). Interactions between *Pseudomonas putida* UW4 and *Gigaspora rosea* BEG9 and their consequences for the growth of cucumber under salt-stress conditions. J. Appl. Microbiol. 108, 236–245. doi: 10.1111/j.1365-2672.2009.04414.x, PMID: 19566717

[ref29] Gamalero E. Glick B. R. (2015). Bacterial modulation of plant ethylene levels. Plant Physiol. 169, 13–22. doi: 10.1104/pp.15.00284, PMID: 25897004 PMC4577377

[ref30] Glick B. (1995). The enhancement of plant growth by free-living bacteria. Can. J. Microbiol. 117, 109–117.

[ref31] Glick B. R. (2012). Plant growth-promoting bacteria: mechanisms and applications. Scientifica. 2012, 1–15. doi: 10.6064/2012/963401, PMID: 24278762 PMC3820493

[ref32] Glick B. R. (2014). Bacteria with ACC deaminase can promote plant growth and help to feed the world. Microbiol. Res. 169, 30–39. doi: 10.1016/j.micres.2013.09.009 24095256

[ref33] Glick B. Penrose D. Li J. (1998). A model for the lowering of plant ethylene concentrations by plant growth-promoting bacteria. J. Theor. Biol. 190, 63–68. doi: 10.1006/jtbi.1997.0532, PMID: 9473391

[ref34] Glickmann E. Dessaux Y. (1995). A critical examination of the specificity of the salkowski reagent for indolic compounds produced by phytopathogenic bacteria. Appl. Environ. Microbiol. 61, 793–796. doi: 10.1128/aem.61.2.793-796.1995, PMID: 16534942 PMC1388360

[ref35] Gopinath S. C. B. Anbu P. Arshad M. K. M. Lakshmipriya T. Voon C. H. Hashim U.. (2017). Biotechnological processes in microbial amylase production. Biomed. Res. Int. 2017:1272193. doi: 10.1155/2017/1272193 28280725 PMC5322433

[ref36] Grant J. R. Stothard P. (2008). The CGView server: a comparative genomics tool for circular genomes. Nucleic Acids Res. 36, W181–W184. doi: 10.1093/nar/gkn179, PMID: 18411202 PMC2447734

[ref37] Großkinsky D. K. Tafner R. Moreno M. V. Stenglein S. A. García de Salamone I. E. Nelson L. M.. (2016). Cytokinin production by *Pseudomonas fluorescens* G20-18 determines biocontrol activity against pseudomonas syringae in Arabidopsis. Sci. Rep. 6:23310. doi: 10.1038/srep23310 26984671 PMC4794740

[ref38] Guinel F. C. (2015). Ethylene, a hormone at the center-stage of nodulation. Front. Plant Sci. 6:1121. doi: 10.3389/fpls.2015.01121, PMID: 26834752 PMC4714629

[ref39] Hoagland D. R. Arnon D. I. (1950). The water-culture method for growing plants without soil. Circ. Calif. Agric. Exp. Stn. 347.

[ref40] Huang H. Ullah F. Zhou D. X. Yi M. Zhao Y. (2019). Mechanisms of ROS regulation of plant development and stress responses. Front. Plant Sci. 10:800. doi: 10.3389/fpls.2019.00800, PMID: 31293607 PMC6603150

[ref41] Jaemsaeng R. Jantasuriyarat C. Thamchaipenet A. (2018). Molecular interaction of 1-aminocyclopropane-1-carboxylate deaminase (ACCD)-producing endophytic *Streptomyces* sp. GMKU 336 towards salt-stress resistance of *Oryza sativa* L. cv. KDML105. Sci. Rep. 8:1950. doi: 10.1038/s41598-018-19799-9, PMID: 29386629 PMC5792428

[ref42] Karnwal A. Kaushik P. (2011). Cytokinin production by fluorescent *Pseudomonas* in the presence of rice root exudates. Arch. Phytopathol. Plant Prot. 44, 1728–1735. doi: 10.1080/03235408.2010.526768

[ref43] Kasana R. C. Salwan R. Dhar H. Dutt S. Gulati A. (2008). A rapid and easy method for the detection of microbial cellulases on agar plates using Gram’s iodine. Curr. Microbiol. 57, 503–507. doi: 10.1007/s00284-008-9276-8, PMID: 18810533

[ref44] Keel C. Weller D. M. Natsch A. Cook R. J. (1996). Conservation of the 2, 4-diacetylphloroglucinol biosynthesis locus among fluorescent Pseudomonas strains from diverse geographic locations. Appl. Environ. Microbiol. 62, 552–563. doi: 10.1128/aem.62.2.552-563.1996, PMID: 8593055 PMC167820

[ref45] Keunen E. Schellingen K. Vangronsveld J. Cuypers A. (2016). Ethylene and metal stress: small molecule, big impact. Front. Plant Sci. 7:23. doi: 10.3389/fpls.2016.00023, PMID: 26870052 PMC4735362

[ref46] Khan M. I. R. Fatma M. Per T. S. Anjum N. A. Khan N. A. (2015). Salicylic acid-induced abiotic stress tolerance and underlying mechanisms in plants. Front. Plant Sci. 6:462. doi: 10.3389/fpls.2015.00462, PMID: 26175738 PMC4485163

[ref47] Kim Y. C. Miller C. D. Anderson A. J. (2000). Superoxide dismutase activity in *Pseudomonas putida* affects utilization of sugars and growth on root surfaces. Appl. Environ. Microbiol. 66, 1460–1467. doi: 10.1128/AEM.66.4.1460-1467.2000, PMID: 10742227 PMC92008

[ref48] Kim H.-E. Shitashiro M. Kuroda A. Takiguchi N. Kato J. (2007). Ethylene chemotaxis in *Pseudomonas aeruginosa* and other *Pseudomonas* species. Microbes Environ. 22, 186–189. doi: 10.1264/jsme2.22.186

[ref49] Kouker G. Jaeger K.-E. (1987). Specific and sensitive plate assay for bacterial lipase. Appl Env. Microb 53, 211–213. doi: 10.1128/aem.53.1.211-213.1987 PMC203632 3103532

[ref50] Lanteigne C. Gadkar V. J. Wallon T. Novinscak A. Filion M. (2012). Production of DAPG and HCN by *Pseudomonas* sp. LBUM300 contributes to the biological control of bacterial canker of tomato. Phytopathology 102, 967–973. doi: 10.1094/PHYTO-11-11-0312, PMID: 22713078

[ref51] Lebeis S. L. Paredes S. H. Lundberg D. S. Breakfield N. Gehring J. McDonald M.. (2015). Salicylic acid modulates colonization of the root microbiome by specific bacterial taxa. Science 349, 860–864. doi: 10.1126/science.aaa8764, PMID: 26184915

[ref52] Li J. Glick B. (2001). Transcriptional regulation of the *Enterobacter cloacae* UW4 1-aminocyclopropane-1-carboxylate (ACC) deaminase gene (acdS). Can. J. Microbiol. 367, 359–367. doi: 10.1139/cjm-47-4-359 11358176

[ref53] Liu Q. Luo L. Zheng L. (2018). Lignins: biosynthesis and biological functions in plants. Int. J. Mol. Sci. 19:335. doi: 10.3390/ijms19020335, PMID: 29364145 PMC5855557

[ref54] Lund S. T. Stall R. E. Klee H. J. (1998). Ethylene regulates the susceptible response to pathogen infection in tomato. Plant Cell 10, 371–382. doi: 10.1105/tpc.10.3.371, PMID: 9501111 PMC144005

[ref55] Luo R. Liu B. Xie Y. Li Z. Huang W. Yuan J.. (2012). SOAPdenovo2: an empirically improved memory-efficient short-read de novo assembler. Gigascience 1:18. doi: 10.1186/2047-217X-1-18, PMID: 23587118 PMC3626529

[ref56] Ma W. Charles T. C. Glick B. R. (2004). Expression of an exogenous 1-aminocyclopropane-1-carboxylate deaminase gene in *Sinorhizobium meliloti* increases its ability to nodulate alfalfa. Appl. Environ. Microbiol. 70, 5891–5897. doi: 10.1128/AEM.70.10.5891-5897.2004, PMID: 15466529 PMC522075

[ref57] Mandryk-Litvinkovich M. N. Muratova A. A. Nosonova T. L. Evdokimova O. V. Valentovich L. N. Titok M. A.. (2017). Molecular genetic analysis of determinants defining synthesis of 2,4-diacetylphloroglucinol by *Pseudomonas brassicacearum* BIM B-446 bacteria. Appl. Biochem. Microbiol. 53, 31–39. doi: 10.1134/S0003683817010124

[ref58] Marin A. M. Souza E. M. Pedrosa F. O. Souza L. M. Sassaki G. L. Baura V. A.. (2013). Naringenin degradation by the endophytic diazotroph *Herbaspirillum seropedicae* SmR1. Microbiology 159, 167–175. doi: 10.1099/mic.0.061135-0, PMID: 23125118

[ref59] Marques A. P. G. C. Pires C. Moreira H. Rangel A. O. S. S. Castro P. M. L. (2010). Assessment of the plant growth promotion abilities of six bacterial isolates using *Zea mays* as indicator plant. Soil Biol. Biochem. 42, 1229–1235. doi: 10.1016/j.soilbio.2010.04.014

[ref60] Matthijs S. Brandt N. Ongena M. Achouak W. Meyer J. M. Budzikiewicz H. (2016). Pyoverdine and histicorrugatin-mediated iron acquisition in *Pseudomonas thivervalensis*. Biometals 29, 467–485. doi: 10.1007/s10534-016-9929-1, PMID: 27007713

[ref61] Mayak S. Tirosh T. Glick B. R. (2004). Plant growth-promoting bacteria that confer resistance to water stress in tomatoes and peppers. Plant Sci. 166, 525–530. doi: 10.1016/j.plantsci.2003.10.025

[ref62] Molina L. Constantinescu F. Michel L. Reimmann C. Duffy B. Défago G. (2003). Degradation of pathogen quorum-sensing molecules by soil bacteria: a preventive and curative biological control mechanism. FEMS Microbiol. Ecol. 45, 71–81. doi: 10.1016/S0168-6496(03)00125-9, PMID: 19719608

[ref63] Murphy P. J. Wexler W. Grzemski W. Rao J. P. Gordon D. (1995). Rhizopines-their role in symbiosis and competition. Soil Biol. Biochem. 27, 525–529. doi: 10.1016/0038-0717(95)98627-Z

[ref64] Nascimento F. X. Espada M. Barbosa P. Rossi M. J. Vicente C. S. L. Mota M. (2016). Non-specific transient mutualism between the plant parasitic nematode, *Bursaphelenchus xylophilus*, and the opportunistic bacterium *Serratia quinivorans* BXF1, a plant-growth promoting pine endophyte with antagonistic effects. Environ. Microbiol. 18, 5265–5276. doi: 10.1111/1462-2920.13568, PMID: 27768814

[ref65] Nascimento F. X. Glick B. R. Rossi M. J. (2019). Isolation and characterization of novel soil- and plant-associated bacteria with multiple phytohormone-degrading activities using a targeted methodology. Access Microbiol. 1. doi: 10.1099/acmi.0.000053, PMID: 32974544 PMC7481731

[ref66] Nascimento F. X. Hernandez A. G. Glick B. R. Rossi M. J. (2020). The extreme plant-growth-promoting properties of *Pantoea phytobeneficialis* MSR2 revealed by functional and genomic analysis. Environ. Microbiol. 22, 1341–1355. doi: 10.1111/1462-2920.14946, PMID: 32077227

[ref67] Nascimento F. X. Rossi M. J. Glick B. R. (2018a). Ethylene and 1-aminocyclopropane-1-carboxylate (ACC) in plant–bacterial interactions. Front. Plant Sci. 9:114. doi: 10.3389/fpls.2018.00114 PMC5827301 29520283

[ref68] Nascimento F. X. Tavares M. J. Rossi M. J. Glick B. R. (2018b). The modulation of leguminous plant ethylene levels by symbiotic rhizobia played a role in the evolution of the nodulation process. Heliyon 4:e01068. doi: 10.1016/j.heliyon.2018.e01068 30603701 PMC6304460

[ref69] Nascimento F. X. Vicente C. S. L. Barbosa P. Espada M. Glick B. R. Mota M.. (2013). Evidence for the involvement of ACC deaminase from *Pseudomonas putida* UW4 in the biocontrol of pine wilt disease caused by *Bursaphelenchus xylophilus*. BioControl 58, 427–433. doi: 10.1007/s10526-012-9500-0

[ref70] Nelkner J. Tejerizo G. T. Hassa J. Lin T. W. Witte J. Verwaaijen B.. (2019). Genetic potential of the biocontrol agent *Pseudomonas brassicacearum* (Formerly P. trivialis) 3Re2-7 unraveled by genome sequencing and mining, comparative genomics and transcriptomics. Genes 10:601. doi: 10.3390/genes10080601, PMID: 31405015 PMC6722718

[ref71] Novinscak A. Gadkar V. J. Joly D. L. Filion M. (2016). Complete genome sequence of *Pseudomonas brassicacearum* LBUM300, a disease-suppressive bacterium with antagonistic activity toward fungal, oomycete, and bacterial plant pathogens. Genome Announc. 4. doi: 10.1128/genomeA.01623-15, PMID: 26823582 PMC4732335

[ref72] Orozco-Mosqueda M. D. C. Duan J. DiBernardo M. Zetter E. Campos-García J. Glick B. R.. (2019). The production of ACC deaminase and trehalose by the plant growth promoting bacterium *Pseudomonas* sp. UW4 synergistically protect tomato plants against salt stress. Front. Microbiol. 10:1392. doi: 10.3389/fmicb.2019.01392, PMID: 31275294 PMC6594411

[ref73] Pillai B. V. S. Swarup S. (2002). Elucidation of the flavonoid catabolism pathway in *Pseudomonas putida* pml2 by comparative metabolic profiling. Appl. Environ. Microbiol. 68, 143–151. doi: 10.1128/AEM.68.1.143-151.2002, PMID: 11772620 PMC126565

[ref74] Pritchard L. Glover R. H. Humphris S. Elphinstone J. G. Toth I. K. (2016). Genomics and taxonomy in diagnostics for food security: soft-rotting enterobacterial plant pathogens. Anal. Methods 8, 12–24. doi: 10.1039/C5AY02550H

[ref75] Rahman Pour R. Bugg T. D. H. (2015). Characterisation of Dyp-type peroxidases from *Pseudomonas fluorescens* Pf-5: oxidation of Mn(II) and polymeric lignin by Dyp1B. Arch. Biochem. Biophys. 574, 93–98. doi: 10.1016/j.abb.2014.12.022 25558792

[ref76] Ramette A. Frapolli M. Défago G. Moënne-Loccoz Y. (2003). Phylogeny of HCN synthase-encoding hcnBC genes in biocontrol fluorescent pseudomonads and its relationship with host plant species and HCN synthesis ability. Mol. Plant-Microbe Interact. 16, 525–535. doi: 10.1094/MPMI.2003.16.6.525, PMID: 12795378

[ref77] Rao J. R. Cooper J. E. (1994). Rhizobia catabolize nod gene-inducing flavonoids via C-ring fission mechanisms. J. Bacteriol. 176, 5409–5413. doi: 10.1128/jb.176.17.5409-5413.1994, PMID: 8071218 PMC196728

[ref78] Rashid S. Charles T. C. Glick B. R. (2012). Isolation and characterization of new plant growth-promoting bacterial endophytes. Appl. Soil Ecol. 61, 217–224. doi: 10.1016/j.apsoil.2011.09.011

[ref79] Redondo-Nieto M. Barret M. Morrissey J. Germaine K. Martínez-Granero F. Barahona E.. (2013). Genome sequence reveals that *Pseudomonas fluorescens* F113 possesses a large and diverse array of systems for rhizosphere function and host interaction. BMC Genomics 14:54. doi: 10.1186/1471-2164-14-54 23350846 PMC3570484

[ref80] Rivas-San Vicente M. Plasencia J. (2011). Salicylic acid beyond defence: its role in plant growth and development. J. Exp. Bot. 62, 3321–3338. doi: 10.1093/jxb/err031, PMID: 21357767

[ref81] Riyazuddin R. Verma R. Singh K. Nisha N. Keisham M. Bhati K. K.. (2020). Ethylene: A master regulator of salinity stress tolerance in plants. Biomol. Ther. 10, 1–22. doi: 10.3390/biom10060959 PMC7355584 32630474

[ref82] Samanovic M. I. Tu S. Novák O. Iyer L. M. McAllister F. E. Aravind L.. (2015). Proteasomal control of cytokinin synthesis protects *Mycobacterium tuberculosis* against nitric oxide. Mol. Cell 57, 984–994. doi: 10.1016/j.molcel.2015.01.024, PMID: 25728768 PMC4369403

[ref83] Saravanakumar D. Samiyappan R. (2007). ACC deaminase from *Pseudomonas fluorescens* mediated saline resistance in groundnut (*Arachis hypogea)* plants. J. Appl. Microbiol. 102, 1283–1292. doi: 10.1111/j.1365-2672.2006.03179.x, PMID: 17448163

[ref84] Schiller D. Kruse D. Kneifel H. Kramer R. Burkovski A. (2000). Polyamine transport and role of potE in response to osmotic stress in *Escherichia coli*. J. Bacteriol. 182, 6247–6249. doi: 10.1128/JB.182.21.6247-6249.2000, PMID: 11029450 PMC94764

[ref85] Schwyn B. Neilands J. B. (1987). Universal chemical assay for the detection and determination of siderophores. Anal. Biochem. 160, 47–56. doi: 10.1016/0003-2697(87)90612-9, PMID: 2952030

[ref86] Sharma S. K. Sharma M. P. Ramesh A. Joshi O. P. (2011). Characterization of zinc-solubilizing bacillus isolates and their potential to influence zinc assimilation in soybean seeds. J. Microbiol. Biotechnol. 22, 352–359. doi: 10.4014/jmb.1106.05063 22450791

[ref87] Singh H. P. Kaur S. Batish D. R. Kohli R. K. (2014). Ferulic acid impairs rhizogenesis and root growth, and alters associated biochemical changes in mung bean (*Vigna radiata*) hypocotyls. J. Plant Interact. 9, 267–274. doi: 10.1080/17429145.2013.820360

[ref88] Sumayo M. Hahm M. S. Ghim S. Y. (2013). Determinants of plant growth-promoting *Ochrobactrum lupini* KUDC1013 involved in induction of systemic resistance against *Pectobacterium carotovorum subsp. carotovorum* in tobacco leaves. Plant Pathol. J. 29, 174–181. doi: 10.5423/PPJ.SI.09.2012.0143, PMID: 25288944 PMC4174773

[ref89] Tao J. J. Chen H. W. Ma B. Zhang W. K. Chen S. Y. Zhang J. S. (2015). The role of ethylene in plants under salinity stress. Front. Plant Sci. 6:1059. doi: 10.3389/fpls.2015.01059 PMC4661241 26640476

[ref90] Taylor J. L. Zaharia L. I. Chen H. Anderson E. Abrams S. R. (2006). Biotransformation of adenine and cytokinins by the rhizobacterium *Serratia proteamaculans*. Phytochemistry 67, 1887–1894. doi: 10.1016/j.phytochem.2006.06.016, PMID: 16860349

[ref91] Tiepo A. N. Constantino L. V. Madeira T. B. Gonçalves L. S. A. Pimenta J. A. Bianchini E.. (2020). Plant growth-promoting bacteria improve leaf antioxidant metabolism of drought-stressed Neotropical trees. Planta 251, 1–11. doi: 10.1007/s00425-020-03373-7 32189086

[ref92] Timmusk S. Paalme V. Pavlicek T. Bergquist J. Vangala A. Danilas T.. (2011). Bacterial distribution in the rhizosphere of wild barley under contrasting microclimates. PLoS One 6:e17968. doi: 10.1371/journal.pone.0017968, PMID: 21448272 PMC3063161

[ref93] Truyens S. Weyens N. Cuypers A. Vangronsveld J. (2013). Changes in the population of seed bacteria of transgenerationally Cd-exposed *Arabidopsis thaliana*. Plant Biol. 15, 971–981. doi: 10.1111/j.1438-8677.2012.00711.x, PMID: 23252960

[ref94] Turner J. A. Rice E. L. (1975). Microbial decomposition of ferulic acid in soil. J. Chem. Ecol. 1, 41–58. doi: 10.1007/BF00987719

[ref95] Vacheron J. Desbrosses G. Ebastien Reoud S. Padilla R. Walker V. Muller D.. (2018). Differential contribution of plant-beneficial functions from *Pseudomonas kilonensis* F113 to root system architecture alterations in *Arabidopsis thaliana* and *Zea mays*. Mol. Plant-Microbe Interact. 31, 212–223. doi: 10.1094/MPMI-07-17-0185-R, PMID: 28971723

[ref96] Van de Poel B. Smet D. Van Der Straeten D. (2015). Ethylene and hormonal cross talk in vegetative growth and development. Plant Physiol. 169, 61–72. doi: 10.1104/pp.15.00724, PMID: 26232489 PMC4577414

[ref97] Vicente C. S. L. Nascimento F. Espada M. Barbosa P. Mota M. Glick B. R.. (2012). Characterization of bacteria associated with pinewood nematode *Bursaphelenchus xylophilu*s. PLoS One 7:e46661. doi: 10.1371/journal.pone.0046661, PMID: 23091599 PMC3473040

[ref98] Xie S. Wu H.-J. Zang H. Wu L. Zhu Q. Gao X. (2014). Plant growth promotion by spermidine-producing *Bacillus subtilis* OKB105. Mol. Plant-Microbe Interact. 27, 655–663. doi: 10.1094/MPMI-01-14-0010-R, PMID: 24678831

[ref99] Zeng Y. F. Ko T. P. Lai H. L. Cheng Y. S. Wu T. H. Ma Y.. (2011). Crystal structures of *Bacillus* alkaline phytase in complex with divalent metal ions and inositol hexasulfate. J. Mol. Biol. 409, 214–224. doi: 10.1016/j.jmb.2011.03.063, PMID: 21463636

