# Supplementary material for: Genomic Analysis of the 1-Aminocyclopropane-1-Carboxylate Deaminase-Producing Pseudomonas thivervalensis SC5 Reveals Its Multifaceted Roles in Soil and in Beneficial Interactions With Plants
Source: Front Microbiol. 2021 Sep 30;12:752288. doi: 10.3389/fmicb.2021.752288 (PMC8515041; doi:10.3389/fmicb.2021.752288)
Supplement: Supplementary file 1 [file Data_Sheet_1.DOCX]

**Genomic analysis of the ACC deaminase-producing *Pseudomonas thivervalensis* SC5 reveals its multifaceted roles in soil and in beneficial interactions with plants**

Francisco X. Nascimento, Paola Urón, Bernard R. Glick, Admir Giachini, Márcio J. Rossi

**SUPPLEMENTARY INFORMATION**

**SUPPLEMENTARY FIGURES**

**
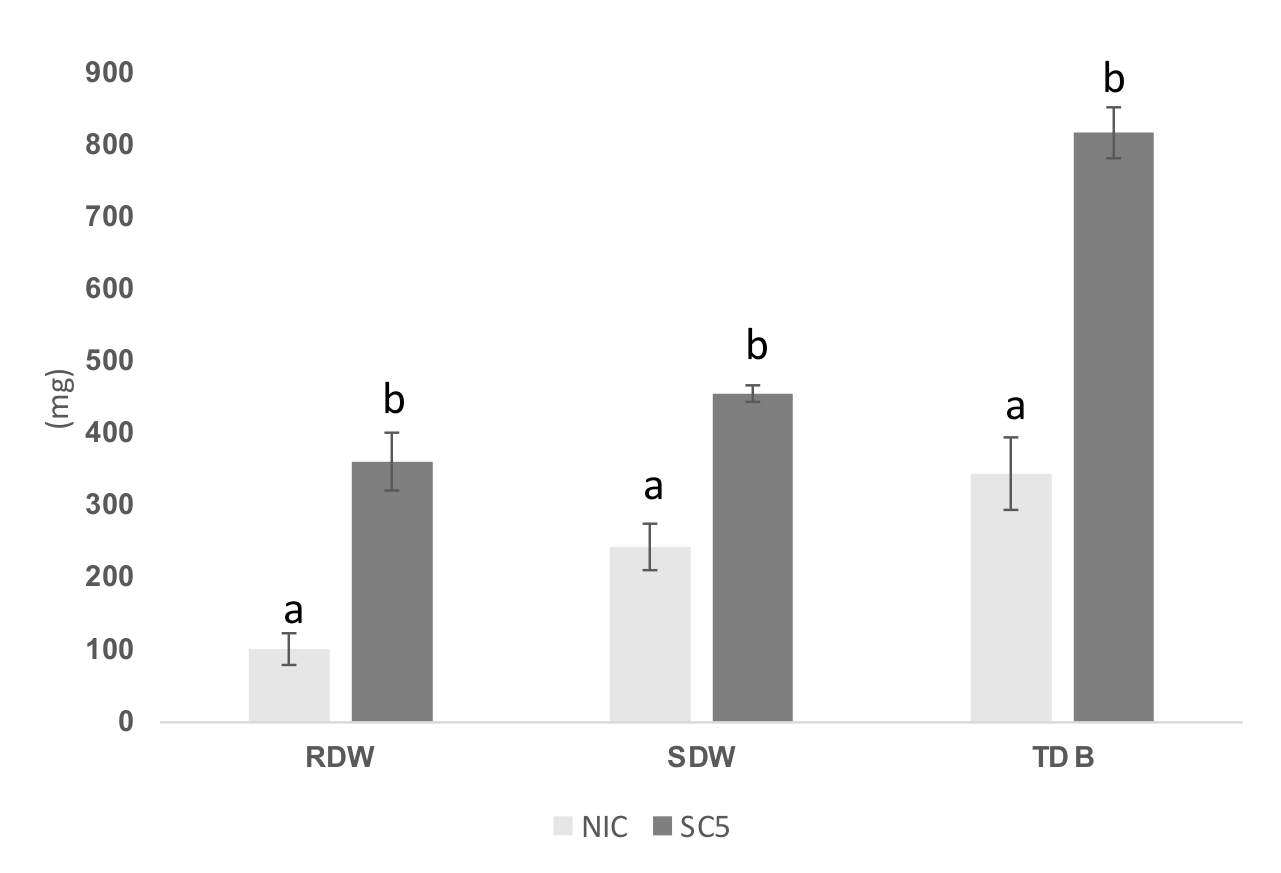
**

**Figure S1**- Results obtained from the cucumber plant growth promotion assay conducted under laboratory conditions, three weeks after inoculation.

RDW- Root Dry Weight; SDW- Shoot Dry Weight; TDB- Total Dry Biomass.

Different letters above the standard error bars represent significant statistical differences (p<0.05) between the treatments in each parameter (RDW, SDW, TDB).


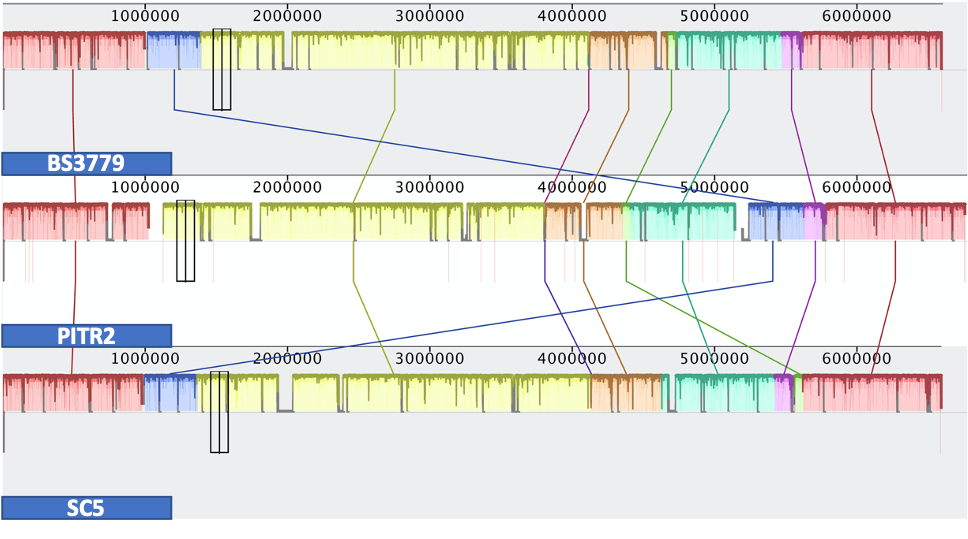


**Figure S2**- Genome alignments between *Pseudomonas* *thivervalensis* strains. Identical colors represent similar genomic organization/genome sections.

**SUPPLEMENTARY TABLES**

**Table S1-** Genes involved in bacterial secretion systems

| **Locus tag** | **Gene** | **Product** | **Pathway** |
| --- | --- | --- | --- |
| **CE140_21695** | *secA* | preprotein translocase subunit SecA | **Sec system** |
| **CE140_17870** | *secB* | preprotein translocase subunit SecB |  |
| **CE140_14795** | *secD* | preprotein translocase subunit SecD |  |
| **CE140_25495** | *secE* | preprotein translocase subunit SecE |  |
| **CE140_14790** | *secF* | preprotein translocase subunit SecF |  |
| **CE140_15565** | *secG* | preprotein translocase subunit SecG |  |
| **CE140_25330** | *secY* | preprotein translocase subunit SecY |  |
| **CE140_14540** | *SRP54* | signal recognition particle subunit SRP5 |  |
| **CE140_26715** | *ftsY* | fused signal recognition particle receptor |  |
| **CE140_14800** | *yajC* | preprotein translocase subunit YajC |  |
| **CE140_28560** | *yidC* | YidC/Oxa1 family membrane |  |
| **CE140_17645** | *tatA* | sec-independent protein translocase protein TatA | **Tat secretion system** |
| **CE140_17650** | *tatB* | sec-independent protein translocase protein TatB |  |
| **CE140_17655** | *tatC* | sec-independent protein translocase protein TatC |  |
| **CE140_04285**  **CE140_06905**  **CE140_17035** | *tolC* | outer membrane protein | **Type I secretion system** |
| **CE140_04510**  **CE140_06915** | *prtD* | ATP-binding cassette, subfamily C, bacterial exporter for protease/lipase | **Hemophore/metalloprotease transporter** |
| **CE140_04515**  **CE140_06910** | *prtE* | membrane fusion protein, protease secretion system |  |
| **CE140_04520** | *prtF* | outer membrane protein, protease secretion system |  |
| **CE140_12200**  **CE140_18655** | *lapB* | ATP-binding cassette, subfamily C, bacterial LapB | **Adhesin protein transporter** |
| **CE140_12205**  **CE140_18650** | *lapC* | membrane fusion protein, adhesin transport system |  |
| **CE140_12195**  **CE140_18660** | *lapE* | outer membrane protein, adhesin transport system |  |
| **CE140_04280** | *eexE* | membrane fusion protein, epimerase transport system | **AlgE-type Mannuronan C-5-Epimerase transporter** |
| **CE140_04275** | *eexD* | ATP-binding cassette, subfamily C, bacterial EexD |  |
| **CE140_03655**  **CE140_17460** | *gspD* | general secretion pathway protein D | **Type 2 secretion system** |
| **CE140_03660**  **CE140_17455**  **CE140_27155** | *gspE* | general secretion pathway protein E |  |
| **CE140_03665**  **CE140_17450** | *gspF* | general secretion pathway protein F |  |
| **CE140_03635**  **CE140_17445** | *gspG* | general secretion pathway protein G |  |
| **CE140_03620**  **CE140_17440** | *gspH* | general secretion pathway protein H |  |
| **CE140_03630**  **CE140_17435** | *gspI* | general secretion pathway protein I |  |
| **CE140_03615**  **CE140_17430** | *gspJ* | general secretion pathway protein J |  |
| **CE140_03640**  **CE140_17425** | *gspK* | general secretion pathway protein K |  |
| **CE140_03645**  **CE140_17420** | *gspL* | general secretion pathway protein L |  |
| **CE140_17415** | *gspM* | general secretion pathway protein M |  |
| **CE140_23705** | *pilD* | leader peptidase (prepilin peptidase) / N-methyltransferase |  |
| **CE140_23705** | *yscC* | type III secretion protein C | **Type 3 secretion system** |
| **CE140_26905** | *yscD* | type III secretion protein D |  |
| **CE140_26970** | *yscJ* | type III secretion protein J |  |
| **CE140_26880** | *yscN* | ATP synthase in type III secretion protein N |  |
| **CE140_26965** | *yscQ* | type III secretion protein Q |  |
| **CE140_26950** | *yscR* | type III secretion protein R |  |
| **CE140_26945** | *yscS* | type III secretion protein S |  |
| **CE140_26940** | *yscT* | type III secretion protein T |  |
| **CE140_26935** | *yscU* | type III secretion protein U |  |
| **CE140_26930** | *yscV* | type III secretion protein V |  |
| **CE140_26975** | *yscW* | type III secretion protein W |  |
| **CE140_03190**  **CE140_27850**  **CE140_27980** | *impL* | type VI secretion system protein ImpL | **Type VI secretion system** |
| **CE140_03185**  **CE140_27855**  **CE140_27985** | *impK* | type VI secretion system protein ImpK |  |
| **CE140_03180**  **CE140_27860**  **CE140_27990** | *impJ* | type VI secretion system protein ImpJ |  |
| **CE140_03145**  **CE140_27915**  **CE140_28020** | *impH* | type VI secretion system protein ImpH |  |
| **CE140_03150**  **CE140_27910**  **CE140_28025** | *impG* | type VI secretion system protein ImpG |  |
| **CE140_03155**  **CE140_27905** | *impF* | type VI secretion system protein ImpF |  |
| **CE140_03165**  **CE140_27885**  **CE140_28035** | *impC* | type VI secretion system protein ImpC |  |
| **CE140_03170**  **CE140_27880**  **CE140_28040** | *impB* | type VI secretion system protein ImpB |  |
| **CE140_03195**  **CE140_27875** | *impA* | type VI secretion system protein ImpA |  |
| **CE140_03160**  **CE140_09340**  **CE140_27890** | *hcp* | type VI secretion system secreted protein Hcp |  |
| **CE140_03130**  **CE140_05275**  **CE140_09870**  **CE140_16310**  **CE140_17200**  **CE140_27925**  **CE140_28050** | *vgrG* | type VI secretion system secreted protein VgrG |  |
| **CE140_28030** |  | type VI secretion system protein |  |
| **CE140_27865**  **CE140_27995** | *vasD* | type VI secretion system protein VasD |  |
| **CE140_03140**  **CE140_27920**  **CE140_28015** | *vasG* | type VI secretion system protein VasG |  |
| **CE140_28045** | *vasJ* | type VI secretion system protein VasJ |  |
| **CE140_27845** | *impM* | type VI secretion system protein ImpM |  |
| **CE140_27870** | *fha1* | type VI secretion system protein |  |
| **CE140_03175** | *lip3* | type VI secretion system protein |  |

**Table S2**- Genes involved in nitrogen metabolism

| **Locus tag** | **Gene** | **Product** | **Pathway** |
| --- | --- | --- | --- |
| **CE140_10880** | *nasD* | Assimilatory nitrite reductase [NAD(P)H] large subunit | **Nitrate reduction** |
| **CE140_10885** | *nasE* | Assimilatory nitrite reductase [NAD(P)H] small subunit |  |
| **CE140_10890** | *nasA* | Assimilatory nitrate reductase catalytic subunit |  |
| **CE140_04680** | *nirB* | Nitrite reductase (NADH) large subunit |  |
| **CE140_04685** | *nirD* | Nitrite reductase (NADH) small subunit |  |
| **CE140_06390** | *narI* | nitrate reductase gamma subunit |  |
| **CE140_06395** | *narW* | nitrate reductase molybdenum cofactor assembly chaperone NarW |  |
| **CE140_06400** | *narH* | nitrate reductase / nitrite oxidoreductase, beta subunit |  |
| **CE140_06405** | *narG* | nitrate reductase / nitrite oxidoreductase, alpha subunit |  |
| **CE140_03945** | *nirG* | Protein NirG | **Denitrification** |
| **CE140_03940** | *nirH* | Protein NirH |  |
| **CE140_03950** | *nirL* | Protein NirL |  |
| **CE140_03955** | *nirD* | Protein NirD |  |
| **CE140_03960** | *nirF* | Protein NirF |  |
| **CE140_03965** | *nirC* | Cytochrome c55X |  |
| **CE140_03970** | *nirM* | Cytochrome c-551 |  |
| **CE140_03975** | *nirS* | Nitrite reductase (NO-forming) |  |
| **CE140_03980** | *nirQ* | Denitrification regulatory protein NirQ |  |
| **CE140_03995** | *norC* | Nitric oxide reductase subunit C |  |
| **CE140_04000** | *norB* | Nitric oxide reductase subunit B |  |
| **CE140_04005** | *norD* | Protein NorD |  |
| **CE140_06845** | *nosZ* | Nitrous-oxide reductase |  |
| **CE140_06410**  **CE140_06415** | *narK* | Nitrate/nitrite transporter NarK | **Nitrate/nitrite transport** |
| **CE140_10870** | *nrtP* | MFS transporter, NNP family, nitrate/nitrite transporter |  |
| **CE140_10860** | *nrtA* | nitrate/nitrite transport system substrate-binding protein |  |
| **CE140_00910**  **CE140_02545**  **CE140_12795**  **CE140_18640** | *ABC.SN.S* | NitT/TauT family transport system substrate-binding protein |  |
| **CE140_00915**  **CE140_01685**  **CE140_12800**  **CE140_18630** | ABC.SN.P | NitT/TauT family transport system permease protein |  |
| **CE140_00920**  **CE140_01680**  **CE140_02540**  **CE140_12805**  **CE140_18635** | ABC.SN.A | NitT/TauT family transport system ATP-binding protein |  |
| **CE140_16570** | *ureC* | Urease subunit alpha | **Urea degradation and transport** |
| **CE140_16565** | *ureB* | Urease subunit beta |  |
| **CE140_16550** | *ureA* | Urease subunit gamma |  |
| **CE140_12825** | *atzF* | Allophanate hydrolase |  |
| **CE140_08435**  **CE140_16520** | *urtA* | Urea transport system substrate-binding protein |  |
| **CE140_16525** | *urtB* | Urea transport system permease protein |  |
| **CE140_16530** | *urtC* | Urea transport system permease protein |  |
| **CE140_16535** | *urtD* | Urea transport system ATP-binding protein |  |
| **CE140_16540** | *urtE* | Urea transport system ATP-binding protein |  |
| **CE140_20115** | *utp* | Urea transporter |  |
| **CE140_08345 CE140_11135**  **CE140_27530** | *amt* | Ammonium transporter, Amt family | **Ammonia transport** |
| **CE140_01800**  **CE140_25580** | *npd* | Nitronate monooxygenase | **Oxidative denitrification of nitronates** |
| **CE140_06440**  **CE140_12375**  **CE140_16610** | *azoR* | FMN-dependent NADH-azoreductase |  |

**Table S3-** Genes involved in sulfur metabolism

| **Locus Tag** | **Gene** | **Product** | **Pathway** |
| --- | --- | --- | --- |
| **CE140_15445** | *cysJ* | sulfite reductase (NADPH) flavoprotein alpha-component | **Assimilatory sulfate reduction** |
| **CE140_08935** | *cysI* | sulfite reductase (NADPH) hemoprotein beta-component |  |
| **CE140_19075** | *cysH* | phosphoadenosine phosphosulfate reductase |  |
| **CE140_18785**  **CE140_20125** | *cysP* | Sulphate transport system substrate-binding protein | **Sulfate transport** |
| **CE140_18780** | *cysU* | Sulfate transport system permease protein |  |
| **CE140_18775** | *cysW* | Sulfate transport system permease protein |  |
| **CE140_18770** | *cysA* | Sulfate transport system ATP-binding protein |  |
| **CE140_16020**  **CE140_23220**  **CE140_28905** | *TC.SULP* | sulfate permease, SulP family |  |
| **CE140_18300** | *tauA* | taurine transport system substrate-binding protein | **Taurine transport and degradation** |
| **CE140_02550**  **CE140_18310** | *tauC* | taurine transport system permease protein |  |
| **CE140_18305** | *tauB* | taurine transport system ATP-binding protein |  |
| **CE140_05380**  **CE140_18315**  **CE140_18645** | *tauD* | Taurine dioxygenase |  |
| **CE140_02655**  **CE140_02660**  **CE140_27100** | *ssuA* | sulfonate transport system substrate-binding protein | **Sulfonate transport and degradation** |
| **CE140_02650**  **CE140_27090** | *ssuC* | sulfonate transport system permease protein |  |
| **CE140_02645**  **CE140_27085** | *ssuB* | sulfonate transport system ATP-binding protein |  |
| **CE140_27095** | *ssuD* | Alkanesulfonate monooxygenase |  |
| **CE140_07010** | *sfnG* | Dimethylsulfone monooxygenase |  |
| **CE140_18340** | *dcyD* | D-cysteine desulfhydrase | **D-cysteine degradation** |
| **CE140_01000**  **CE140_01015** | *dddp* | Dimethlysulfonioproprionate lyase DddP homolog | **DMSP degradation** |
| **CE140_07635**  **CE140_18375** | *dmoA* | Dimethyl-sulfide monooxygenase | **DMS degradation** |

**Table S4-** Genes involved in phosphate solubilization and phosphorous acquisition

| **Locus tag** | **Gene** | **Product** | **Pathway** |
| --- | --- | --- | --- |
| **CE140_25845** | *pqqA* | pyrroloquinoline quinone precursor peptide | **PQQ biosynthesis** |
| **CE140_25850** | *pqqB* | pyrroloquinoline-quinone synthase |  |
| **CE140_25855** | *pqqC* | pyrroloquinoline-quinone synthase |  |
| **CE140_25860** | *pqqD* | pyrroloquinoline quinone biosynthesis peptide chaperone |  |
| **CE140_25865** | *pqqE* | Coenzyme PQQ synthesis protein E |  |
| **CE140_25840** | *pqqF* | pyrroloquinoline quinone biosynthesis protein F |  |
| **CE140_22095** | *gcd* | quinoprotein glucose dehydrogenase | **D-gluconate production** |
| **CE140_04710** | *phyC* | 3-phytase | **Organic phosphate solubilization** |
| **CE140_27315** | *ppx* | Exopolyphosphatase |  |
| **CE140_15450**  **CE140_17175** | *phoD* | alkaline phosphatase |  |
| **CE140_02425** | *phnC* | phosphonate transport system ATP-binding protein | **Degradation of phosphonates** |
| **CE140_02430** | *phnD* | phosphonate transport system substrate-binding protein |  |
| **CE140_02415**  **CE140_02420** | *phnE* | phosphonate transport system permease protein |  |
| **CE140_10425**  **CE140_28695** | *phnX* | phosphonoacetaldehyde hydrolase |  |
| **CE140_03675**  **CE140_05000**  **CE140_09315**  **CE140_29225** | *pstS* | phosphate transport system substrate-binding protein | **Phosphate transport** |
| **CE140_04995**  **CE140_29230** | *pstC* | phosphate transport system permease protein |  |
| **CE140_04990**  **CE140_29235** | *pstA* | phosphate transport system permease protein |  |
| **CE140_04985**  **CE140_29240** | *pstB* | phosphate transport system ATP-binding protein |  |
| **CE140_28865** | *yjbB* | phosphate:Na+ symporter |  |

**Table S5**- Genes involved in iron transport and siderophore biosynthesis.

| **Locus tag** | **Gene** | **Product** | **Pathway** |
| --- | --- | --- | --- |
| **CE140_27190**  **CE140_28690** | *fbpA* | iron(III) transport system substrate-binding protein | **Iron(III) transport** |
| **CE140_27185**  **CE140_28700** | *fbpB* | iron(III) transport system permease protein |  |
| **CE140_21390**  **CE140_28705** | *fbpC* | iron(III) transport system ATP-binding protein |  |
| **CE140_06355**  **CE140_27350** | *efeU* | high-affinity iron transporter | **Iron(II) transport** |
| **CE140_24300** | *fieF* | Ferrous-iron efflux pump FieF |  |
| **CE140_06345** | *ftrA* | Periplasmic iron binding protein |  |
| **CE140_07460**  **CE140_16500**  **CE140_23590**  **CE140_25095** | *ABC.FEV.S* | iron complex transport system substrate-binding protein | **Iron complex transport** |
| **CE140_07465**  **CE140_16505**  **CE140_23585** | *ABC.FEV.P* | iron complex transport system permease protein |  |
| **CE140_07470**  **CE140_16495**  **CE140_23580** | *ABC.FEV.A* | iron complex transport system ATP-binding protein |  |
| **CE140_00225**  **CE140_22905** | *fecA* | Fe(3+) dicitrate transport protein |  |
| **CE140_26585**  **CE140_27775** | *tonB* | periplasmic protein TonB |  |
| **CE140_00855**  **CE140_01610** | *fhuE* | outer-membrane receptor for ferric coprogen and ferric-rhodotorulic acid |  |
| **CE140_00115**  **CE140_02905**  **CE140_03880**  **CE140_04770**  **CE140_05160**  **CE140_05165**  **CE140_05975**  **CE140_07435**  **CE140_07455**  **CE140_08460**  **CE140_14865**  **CE140_16485**  **CE140_18745**  **CE140_19915**  **CE140_23150**  **CE140_25995**  **CE140_29025** | *TC.FEV.OM* | iron complex outermembrane receptor protein |  |
| **CE140_00085-90** | *Pyoverdine cluster* | Pyoverdine PYOthi biosynthesis | **Siderophore biosynthesis** |
| **CE140_07030-60** | *Histicorrugatin cluster* | Histicorrugatin biosynthesis and transport |  |
| **CE140_5080-5250** | *Unknown Siderophore cluster* | Siderophore biosynthesis and transport genes |  |

**Table S6-** Genes involved in osmolyte biosynthesis and transport

| **Locus tag** | **Gene** | **Product** | **Pathway** |
| --- | --- | --- | --- |
| **CE140_00865**  **CE140_26260** | *betA* | choline dehydrogenase | **Betaine/Glycine-betaine synthesis** |
| **CE140_21925**  **CE140_26255** | *betB* | Betaine aldehyde dehydrogenase |  |
| **CE140_28895** | *betC* | choline-sulfatase |  |
| **CE140_18335**  **CE140_26245** | *betT* | Choline/glycine/proline betaine transport protein | **Glycine betaine/proline transport system** |
| **CE140_05360** | *proP* | MFS transporter, MHS family, proline/betaine transporter |  |
| **CE140_00990**  **CE140_01035**  **CE140_02295**  **CE140_17700**  **CE140_17735**  **CE140_20055**  **CE140_26190**  **CE140_26230**  **CE140_28900** | *proX* | glycine betaine/proline transport system substrate-binding protein |  |
| **CE140_01040**  **CE140_08990**  **CE140_17730**  **CE140_26235** | *proW* | glycine betaine/proline transport system permease protein |  |
| **CE140_01045**  **CE140_08995**  **CE140_17725**  **CE140_26240** | *proV* | glycine betaine/proline transport system ATP-binding protein |  |
| **CE140_15415** | *opuC* | osmoprotectant transport system substrate-binding protein | **Osmoprotectant transport system** |
| **CE140_15410**  **CE140_15420** | *opuBD* | osmoprotectant transport system permease protein |  |
| **CE140_15425** | *opuA* | osmoprotectant transport system ATP-binding |  |
| **CE140_24495** | *proA* | glutamate-5-semialdehyde dehydrogenase | **Proline biosynthesis and transport** |
| **CE140_23915** | *proB* | glutamate 5-kinase |  |
| **CE140_08980**  **CE140_26635** | *proC* | pyrroline-5-carboxylate reductase |  |
| **CE140_17480** | *gltD* | Glutamate synthase [NADPH] small chain | **Glutamate biosynthesis and transport** |
| **CE140_17485** | *gltB* | Glutamate synthase [NADPH] large chain |  |
| **CE140_08350**  **CE140_22015** | *glxD* | Glutamate synthase large subunit-like protein |  |
| **CE140_21425**  **CE140_26480** | *gltI* | glutamate/aspartate transport system substrate-binding protein |  |
| **CE140_21435** | *gltK* | glutamate/aspartate transport system permease protein |  |
| **CE140_21430** | *gltJ* | glutamate/aspartate transport system permease protein |  |
| **CE140_21440** | *gltL* | glutamate/aspartate transport system ATP-binding protein |  |
| **CE140_08365**  **CE140_09715**  **CE140_12570**  **CE140_17830**  **CE140_20585**  **CE140_27060**  **CE140_27070** | *glnA* | glutamine synthetase | **Glutamine biosynthesis and transport** |
| **CE140_17160** | *glnE* | Bifunctional glutamine synthetase adenylyltransferase/adenylyl-removing enzyme |  |
| **CE140_08855** | *glnP* | glutamine transport system permease protein |  |
| **CE140_07745** | *glnQ* | glutamine transport system ATP-binding protein |  |
| **CE140_11735** | - | N-acetylglutaminylglutamine amidotransferase | **NAGGN biosynthesis** |
| **CE140_11740** | ngg | N-acetylglutaminylglutamine synthetase |  |
| **CE140_11745** | - | osmoprotectant NAGGN system M42 family peptidase |  |
| **CE140_05605** | glgA | glycogen synthase | **Glycogen biosynthesis** |
| **CE140_09485** | *glgB* | 1,4-alpha-glucan branching enzyme |  |
| **CE140_09495** | *glgE* | starch synthase (maltosyl-transferring) |  |
| **CE140_04665**  **CE140_09490** | *treS* | Trehalose synthase/amylase TreS | **Trehalose synthesis** |
| **CE140_05620** | *treY* | (1->4)-alpha-D-glucan 1-alpha-D-glucosylmutase |  |
| **CE140_05610** | *treZ* | maltooligosyltrehalose trehalohydrolase |  |
| **CE140_07180** | *thuE* | trehalose/maltose transport system substrate-binding protein | **Trehalose transport** |
| **CE140_07185** | *thuF* | trehalose/maltose transport system permease protein |  |
| **CE140_07190** | *thuG* | trehalose/maltose transport system permease protein |  |
| **CE140_04140**  **CE140_07195** | *thuK* | multiple sugar transport system ATP-binding protein |  |
| **CE140_22740** | *doeA* | ectoine hydrolase | **Ectoine biosynthesis and transport** |
| **CE140_22735** | *doeB* | N2-acetyl-L-2,4-diaminobutanoate deacetylase |  |
| **CE140_22720** | *doeC* | L-2,4-diaminobutyrate transaminase |  |
| **CE140_22725** | *doeD* | aspartate-semialdehyde dehydrogenase |  |
| **CE140_22760-75** | *ehuABCD* | Ectoine transport system |  |
| **CE140_13590** | *nhaA* | Na+:H+ antiporter, NhaA family | **Sodium and Chloride transport** |
| **CE140_06525** | *nhaB* | Na+:H+ antiporter, NhaB family |  |
| **CE140_03485**  **CE140_03490**  **CE140_18860** | *yfbK* | Ca-activated chloride channel homolog |  |
| **CE140_29175** | *glnT* | sodium/glutamine symporter GlnT |  |
| **CE140_17210** | *putP* | sodium/proline symporter |  |

**Table S7-** Genes involved in polyamines and GABA metabolism and transport.

| **Locus tag** | **Gene** | **Product** | **Pathway** |
| --- | --- | --- | --- |
| **CE140_16315** | *speA* | arginine decarboxylase | **Putrescine biosynthesis** |
| **CE140_18135** | *aguA* | agmatine deiminase |  |
| **CE140_08440** | *aguB* | N-carbamoylputrescine amidase |  |
| **CE140_15430** | *speC* | ornithine decarboxylase |  |
| **CE140_00810** | *hss* | homospermidine synthase | **Spermidine biosynthesis** |
| **CE140_02445** | *cansD* | Carboxynorspermidine synthase |  |
| **CE140_02450** | *nspC* | Carboxynorspermidine/carboxyspermidine decarboxylase |  |
| **CE140_27070** | *puuA* | Gamma-glutamylputrescine synthetase | **Putrescine degradation, conversion to GABA** |
| **CE140_00495**  **CE140_06120**  **CE140_07685**  **CE140_27635** | *puuB* | Gamma-glutamylputrescine oxidoreductase |  |
| **CE140_01020** | *puuC, aldH* | 4-(gamma-glutamylamino)butanal dehydrogenase |  |
| **CE140_27065** | *puuD* | Gamma-glutamyl-gamma-aminobutyrate hydrolase |  |
| **CE140_07325**  **CE140_27055** | *spuC* | putrescine---pyruvate transaminase |  |
| **CE140_14420**  **CE140_14445** | *prr* | Gamma-aminobutyraldehyde dehydrogenase |  |
| **CE140_05065**  **CE140_18825**  **CE140_23070** | *gabT* | 4-aminobutyrate aminotransferase /  (S)-3-amino-2-methylpropionate transaminase | **GABA metabolism** |
| **CE140_05090**  **CE140_08165**  **CE140_18830** | *gabD* | succinate-semialdehyde dehydrogenase /  glutarate-semialdehyde dehydrogenase |  |
| **CE140_04320** | *puuE* | 4-aminobutyrate aminotransferase |  |
| **CE140_06930**  **CE140_27045**  **CE140_27050** | *potF* | putrescine transport system substrate-binding protein | **Putrescine transport** |
| **CE140_27030** | *potI* | putrescine transport system permease protein |  |
| **CE140_27035** | *potH* | putrescine transport system permease protein |  |
| **CE140_27040** | *potG* | putrescine transport system ATP-binding protein |  |
| **CE140_05940**  **CE140_06260**  **CE140_09860**  **CE140_14425**  **CE140_20335**  **CE140_21920**  **CE140_25670** | ABC.SP.S | putative spermidine/putrescine transport system substrate-binding protein | **Polyamine transport** |
| **CE140_05950**  **CE140_09840**  **CE140_14440**  **CE140_20350**  **CE140_21910**  **CE140_25660** | ABC.SP.P | putative spermidine/putrescine transport system permease protein |  |
| **CE140_05945**  **CE140_09835**  **CE140_14435**  **CE140_20345**  **CE140_21915**  **CE140_25665** | ABC.SP.P1 | putative spermidine/putrescine transport system permease protein |  |
| **CE140_05955**  **CE140_09830**  **CE140_14430**  **CE140_20355**  **CE140_21905**  **CE140_25675** | ABC.SP.A | putative spermidine/putrescine transport system ATP-binding protein |  |
| **CE140_06920** | *gabP* | GABA permease | **GABA transport** |

**Table S8**-Genes involved in motility, chemotaxis and attachment

| **Locus tag** | **Gene** | **Product** | **Pathway** |
| --- | --- | --- | --- |
| **CE140_01720**  **CE140_08510**  **CE140_11875** | *cheA* | two-component system, chemotaxis family, sensor kinase CheA | **Chemotaxis two component system** |
| **CE140_01710**  **CE140_08495**  **CE140_08500**  **CE140_11845**  **CE140_11850** | *cheW* | purine-binding chemotaxis protein CheW |  |
| **CE140_01700**  **CE140_08480** | *cheD* | chemotaxis protein CheD |  |
| **CE140_01705**  **CE140_04180**  **CE140_08485**  **CE140_20550** | *cheR* | chemotaxis protein methyltransferase CheR |  |
| **CE140_01695**  **CE140_04185**  **CE140_08475**  **CE140_11870** | *cheB* | two-component system, chemotaxis family, protein-glutamate methylesterase/glutaminase |  |
| **CE140_05470** | *cheBR* | CheB/CheR fusion protein |  |
| **CE140_01730**  **CE140_08520**  **CE140_11885** | *cheY* | two-component system, chemotaxis family, chemotaxis protein CheY |  |
| **CE140_11880** | *cheZ* | chemotaxis protein CheZ |  |
| **CE140_11060**  **CE140_20555**  **CE140_22865** | *cheV* | two-component system, chemotaxis family, chemotaxis protein CheV |  |
| **CE140_00290**  **CE140_01070**  **CE140_01715**  **CE140_01735**  **CE140_01940**  **CE140_02270**  **CE140_02670**  **CE140_05430**  **CE140_05580**  **CE140_05990**  **CE140_06465**  **CE140_07340**  **CE140_08060**  **CE140_08445**  **CE140_08465**  **CE140_08490**  **CE140_08505**  **CE140_08525**  **CE140_08530**  **CE140_11390**  **CE140_12630**  **CE140_15080**  **CE140_15480**  **CE140_15975**  **CE140_16340**  **CE140_16830**  **CE140_17235**  **CE140_17665**  **CE140_17780**  **CE140_18075**  **CE140_18730**  **CE140_19830**  **CE140_21105**  **CE140_23390**  **CE140_23415**  **CE140_23760**  **CE140_24240** | *mcp* | methyl-accepting chemotaxis protein | **MCPs** |
| **CE140_27560** | aer | aerotaxis receptor |  |
| **CE140_11865**  **CE140_16915** | *motA* | chemotaxis protein MotA | **Flagella motor switch** |
| **CE140_11860**  **CE140_16910** | *motB* | chemotaxis protein MotB |  |
| **CE140_21335** | *motY* | sodium-type flagellar protein MotY |  |
| **CE140_11890-1270**  **CE140_20530-20570** | *fli* and *flg* | Flagella biosynthesis operons | **Flagella biosynthesis** |
| **CE140_26555 -26625** | *pil* | twitching motility | **Twitching motility, pilus and chemosensory pili system proteins** |
| **CE140_17510-30**  **CE140_23690-705** | *pil* | type IV pilus assembly |  |
| **CE140_19720-60** | *cpa* | pilus assembly |  |
| **CE140_23860 CE140_23865** | *fimT* | type IV fimbrial biogenesis protein FimT | **Fimbria biogenesis** |
| **CE140_08575** | *bcsQ* | Cellulose biosynthesis protein BcsQ | **Cellulose biosynthesis** |
| **CE140_08570** | *bcsA* | Cellulose synthase catalytic subunit [UDP-forming] |  |
| **CE140_08565** | *bcsB* | Cyclic di-GMP-binding protein |  |
| **CE140_08555** | *bcsC* | Cellulose synthase operon protein C |  |
| **CE140_08560** | *bcsZ* | endoglucanase |  |
| **CE140_08595** | *bcsE* | Cellulose biosynthesis protein BcsE |  |
| **CE140_08585** | *bcsG* | Cellulose biosynthesis protein BcsG |  |
| **CE140_22400-455** | *alg* | Alginate biosynthesis operon | **Alginate biosynthesis** |
| **CE140_09025-100** | *eps* | saccharide biosynthesis cluster | **EPS production** |
| **CE140_09375-9480** | *eps* | saccharide biosynthesis cluster |  |
| **CE140_11475-575** | *eps* | saccharide biosynthesis cluster |  |
| **CE140_28315-90** | *eps* | saccharide biosynthesis cluster |  |
| **CE140_17040-155** | *lps* | lipopolysaccharide biosynthesis cluster | **LPS biosynthesis** |

**Table S9-** Genes involved in oxidative stress response.

| **Locus tag** | **Gene** | **Product** | **Pathway** |
| --- | --- | --- | --- |
| **CE140_09810**  **CE140_24060**  **CE140_25295**  **CE140_28735** | *katE* | catalase | **Resistance to ROS** |
| **CE140_15960** | *katG* | catalase-peroxidase |  |
| **CE140_13185**  **CE140_15190** | *sod* | superoxide dismutase, Fe-Mn family |  |
| **CE140_01865**  **CE140_21355** | *ahpC* | peroxiredoxin  (alkyl hydroperoxide reductase subunit C) |  |
| **CE140_12680** | *bcp* | peroxiredoxin Q/BCP |  |
| **CE140_05785** | *tpx* | atypical 2-Cys peroxiredoxin |  |
| **CE140_22000** |  | cytochrome c peroxidase |  |
| **CE140_11195**  **CE140_23105**  **CE140_24820** | *gpx* | glutathione peroxidase |  |
| **CE140_01110**  **CE140_01580**  **CE140_02865**  **CE140_29015** | *cpo* | non-heme chloroperoxidase |  |
| **CE140_19430**  **CE140_19665**  **CE140_21685**  **CE140_27440**  **CE140_28395** | *gst* | glutathione S-transferase |  |
| **CE140_11270**  **CE140_28660**  **CE140_29325** | *osmC* | lipoyl-dependent peroxiredoxin |  |
| **CE140_09875** | *cybB* | superoxide oxidase |  |
| **CE140_17290-410** | Arylpolyene cluster | Arylpolyene biosynthesis |  |

**Table S10**- Genes involved in carbohydrate metabolism and transport.

| **Locus tag** | **Gene** | **Product** | **Pathway** |
| --- | --- | --- | --- |
| **CE140_06535**  **CE140_13355** | *zwf* | Glucose-6-phosphate 1-dehydrogenase | **Pentose phosphate** |
| **CE140_13350** | *pgl* | 6-phosphogluconolactonase |  |
| **CE140_06540** | *gnd* | 6-phosphogluconate dehydrogenase |  |
| **CE140_25650** | *rpe* | ribulose-phosphate 3-epimerase |  |
| **CE140_10405** | *tal* | Transaldolase |  |
| **CE140_26365** | *tkt* | Transketolase |  |
| **CE140_04470** | *pgm* | phosphoglucomutase, alpha-D-glucose phosphate-specific |  |
| **CE140_27700** | *pmm-pgm* | phosphomannomutase / phosphoglucomutase |  |
| **CE140_23335** | *prsA* | ribose-phosphate pyrophosphokinase |  |
| **CE140_13410** | *glk* | glucokinase | **EMP**  **Glycolysis** |
| **CE140_23660** | *gpi* | Glucose-6-phosphate isomerase |  |
| **CE140_15460** | *fruK* | 1-phosphofructokinase |  |
| **CE140_26340** | *fbaA* | Fructose-bisphosphate aldolase class II |  |
| **CE140_17790** | *fbaB* | Fructose-bisphosphate aldolase class I |  |
| **CE140_15570** | *tpiA* | Triosephosphate isomerase |  |
| **CE140_07805**  **CE140_10495**  **CE140_13420** | *gapA* | Glyceraldehyde-3-phosphate dehydrogenase |  |
| **CE140_26355** | *pgk* | Phosphoglycerate kinase |  |
| **CE140_17885** | *gpmI* | 2,3-bisphosphoglycerate-independent phosphoglycerate mutase |  |
| **CE140_14070** | *eno* | enolase |  |
| **CE140_20110**  **CE140_21820** | *pyk* | pyruvate kinase |  |
| **CE140_22095** | *gcd* | quinoprotein glucose dehydrogenase | **Glucose transformation to gluconate** |
| **CE140_00065** | *gdh* | glucose 1-dehydrogenase |  |
| **CE140_09205** | *rbsK* | ribokinase | **Ribose degradation** |
| **CE140_09200** | *rbsD* | D-ribose pyranase |  |
| **CE140_15460** | *fruK* | 1-phosphofructokinase |  |
| **CE140_03055** | *xylA* | xylose isomerase | **Xylose degradation** |
| **CE140_04130** | *xylB* | xylulokinase |  |
| **CE140_02100**  **CE140_19470** | *xylC* | xylono-1,5-lactonase |  |
| **CE140_07380** | *xylD* | xylonate dehydratase |  |
| **CE140_03430** | *aldH* | 2,5-dioxopentanoate dehydrogenase |  |
| **CE140_15305** | *xylA* | alpha-ketoglutaric semialdehyde dehydrogenase |  |
| **CE140_02080**  **CE140_09555** | *galM* | Aldose 1-epimerase | **Hexose metabolism** |
| **CE140_03100** | *gal* | D-galactose 1-dehydrogenase | **D-galactose degradation** |
| **CE140_05615** | *malQ* | 4-alpha-glucanotransferase | **Maltose degradation** |
| **CE140_04660** | *malZ* | alpha-glucosidase |  |
| **CE140_07200** | *sacA* | Sucrose-6-phosphate hydrolase | **Sucrose degradation** |
| **CE140_24640** | *sacB* | levansucrase |  |
| **CE140_04125** | *scrK* | fructokinase |  |
| **CE140_22455** | *manA* | Mannose-6-phosphate isomerase | **Mannose degradation** |
| **CE140_07120** | *treA* | alpha,alpha-trehalase | **Trehalose degradation** |
| **CE140_21410** | *glpK* | glycerol kinase | **Glycerol degradation** |
| **CE140_13835** | *dgkA* | diacylglycerol kinase |  |
| **CE140_14780** | *suhB* | Inositol-1-monophosphatase | **Myo-inositol degradation** |
| **CE140_02215**  **CE140_06305** | *iolE* | myo-inosose-2 dehydratase |  |
| **CE140_02240**  **CE140_07650** | *iolG* | Inositol 2-dehydrogenase |  |
| **CE140_02230** | *iolI* | 2-keto-myo-inositol isomerase |  |
| **CE140_02235** | *iolD* | 3D-(3,5/4)-trihydroxycyclohexane-1,2-dione acylhydrolase (decyclizing) |  |
| **CE140_02210** | *iolC* | 5-dehydro-2-deoxygluconokinase |  |
| **CE140_02220** | *iolB* | 5-deoxy-glucuronate isomerase |  |
| **CE140_02225**  **CE140_16065**  **CE140_16115** | *mmsA* | methylmalonate-semialdehyde dehydrogenase (CoA acylating) |  |
| **CE140_04135** | *mtlK* | mannitol 2-dehydrogenase | **Mannitol degradation** |
| **CE140_06670** | *sorbD* | galactitol 2-dehydrogenase | **Galactitol/sorbitol degradation** |
| **CE140_13250** | *gntK* | gluconokinase | **D-gluconate degradation** |
| **CE140_03530**  **CE140_07165**  **CE140_08825** | *kdgK* | 2-dehydro-3-deoxygluconokinase | **2KG to 2-keto-6-phosphogluconate** |
| **CE140_05050** | *kduD* | 2-dehydro-3-deoxy-D-gluconate 5-dehydrogenase | **2KG to 6-phospho-D-gluconate** |
| **CE140_03520**  **CE140_22880** | *ghrB* | glyoxylate/hydroxypyruvate/2-ketogluconate reductase | **2KG to 6-phospho-D-gluconate** |
| **CE140_13345** | *eda* | KHG/KDPG aldolase | **KDPG degradation** |
| **CE140_07155** | *gulDH* | L-gulonate dehydrogenase | **L-gulonate degradation to D-fructuronate** |
| **CE140_07140** | *uxuA* | mannonate dehydratase | **D-fructuronate, D-mannonate degradation** |
| **CE140_07145** | *uxuB* | D-mannonate oxidoreductase |  |
| **CE140_07160** | *manD* | mannonate dehydratase |  |
| **CE140_15700** | *dld* | D-lactate dehydrogenase (quinone) | **Lactate degradation** |
| **CE140_15685** | *lldE* | L-lactate dehydrogenase complex protein LldE |  |
| **CE140_15690** | *lldF* | L-lactate dehydrogenase complex |  |
| **CE140_15695** | *lldG* | L-lactate dehydrogenase complex protein LldG |  |
| **CE140_15295** | *garD* | Galactarate dehydratase | **D-galactarate degradation** |
| **CE140_20100** | *garR* | 2-hydroxy-3-oxopropionate reductase |  |
| **CE140_01935** | *garK* | glycerate 2-kinase 1 |  |
| **CE140_03915**  **CE140_09480** | *gudD* | glucarate dehydratase | **D-glucarate degradation** |
| **CE140_28455** | *talrD-galrD* | L-talarate/galactarate dehydratase | **L-talarate degradation** |
| **CE140_19415** | *dgoD* | galactonate dehydratase | **D-galactonate degradation** |
| **CE140_19420** | *dgoA* | 2-dehydro-3-deoxy-6-phosphogalactonate aldolase |  |
| **CE140_19425** | *dgoK* | 2-dehydro-3-deoxygalactonokinase DgoK1 |  |
| **CE140_12980** | *bglX* | Periplasmic beta-glucosidase BglX | **Degradation of**  **Beta-Glucosides** |
| **CE140_13390** | *gstA* | glucose/mannose transport system substrate-binding protein | **Glucose/mannose transporter** |
| **CE140_13385** | *gstB* | glucose/mannose transport system permease protein |  |
| **CE140_13380** | *gstC* | glucose/mannose transport system permease protein |  |
| **CE140_19460** | *araF* | L-arabinose transport system substrate-binding protein | **L-arabinose transport** |
| **CE140_19450** | *araH* | L-arabinose transport system permease protein |  |
| **CE140_19455** | *araG* | L-arabinose transport system ATP-binding protein |  |
| **CE140_03050** | *xylF* | D-xylose transport system substrate-binding protein | **D-Xylose transport** |
| **CE140_03040** | *xylH* | D-xylose transport system permease protein |  |
| **CE140_03045** | *xylG* | D-xylose transport system ATP-binding protein |  |
| **CE140_07730**  **CE140_07890**  **CE140_09225** | *rbsB* | ribose transport system substrate-binding protein | **Ribose transport** |
| **CE140_09215** | *rbsC* | ribose transport system permease protein |  |
| **CE140_09220** | *rbsA* | ribose transport system ATP-binding protein |  |
| **CE140_07180** | *thuE* | trehalose/maltose transport system substrate-binding protein | **Trehalose/maltose transport** |
| **CE140_07185** | *thuF* | trehalose/maltose transport system permease protein |  |
| **CE140_07190** | *thuG* | trehalose/maltose transport system permease protein |  |
| **CE140_04140**  **CE140_07195** | *thuK* | multiple sugar transport system ATP-binding protein |  |
| **CE140_07175** | *scrY* | Sucrose porin | **Sucrose transport** |
| **CE140_04140**  **CE140_07195** | *malK* | multiple sugar transport system ATP-binding protein | **Multiple sugars transport** |
| **CE140_13375** | *msmX* | multiple sugar transport system ATP- binding protein |  |
| **CE140_04155** | *smoE* | sorbitol/mannitol transport system substrate-binding protein | **Sorbitol/mannitol transport** |
| **CE140_04150** | *smoF* | sorbitol/mannitol transport system permease protein |  |
| **CE140_04145** | *smoG* | sorbitol/mannitol transport system permease protein |  |
| **CE140_02250**  **CE140_07660** | *mocB* | inositol transport system permease protein | **Inositol/rhizopine transport** |
| **CE140_02260** | *-* | inositol transport system ATP-binding protein |  |
| **CE140_02255** | *iolT* | MFS transporter, SP family, major inositol transporter |  |
| **CE140_00730** | *glpV* | glycerol transport system substrate-binding protein | **Glycerol transport** |
| **CE140_00715** | *glpP* | glycerol transport system permease protein |  |
| **CE140_00720** | *glpQ* | glycerol transport system permease protein |  |
| **CE140_00705** | *glpS* | glycerol transport system ATP-binding protein |  |
| **CE140_00710** | *glpT* | glycerol transport system ATP-binding protein |  |
| **CE140_18465** | *ptsP* | phosphotransferase system, enzyme I | **Phosphotransferase System**  **Enzyme I** |
| **CE140_15180** | *ptsH* | phosphocarrier protein |  |
| **CE140_15465**  **CE140_22185** | *fruB* | phosphocarrier protein FPr |  |
| **CE140_15455** | *fruA* | PTS system, fructose-specific IIB component | **Fructose specific II component** |

**Table S11-** Genes involved in the metabolism and transport of organic acids.

| **Locus tag** | **Gene** | **Product** | **Pathway** |
| --- | --- | --- | --- |
| **CE140_10625** | *acnA* | aconitate hydratase | **TCA cycle** |
| **CE140_03220** | *acnB* | aconitate hydratase 2 / 2-methylisocitrate dehydratase |  |
| **CE140_01265**  **CE140_01275** | *icd* | Isocitrate dehydrogenase [NADP] |  |
| **CE140_20015** | *sucA* | 2-oxoglutarate dehydrogenase E1 component |  |
| **CE140_20010** | *sucB* | 2-oxoglutarate dehydrogenase E2 component |  |
| **CE140_02045**  **CE140_20005**  **CE140_28310** | *pdhD* | dihydrolipoamide dehydrogenase |  |
| **CE140_20000** | *sucC* | succinyl-CoA synthetase beta subunit |  |
| **CE140_19995** | *sucD* | succinyl-CoA synthetase alpha subunit |  |
| **CE140_20025** | *sdhA* | succinate dehydrogenase/fumarate reductase, flavoprotein subunit |  |
| **CE140_20020** | *sdhB* | succinate dehydrogenase / fumarate reductase, iron-sulfur subunit |  |
| **CE140_20035** | *sdhC* | Succinate dehydrogenase cytochrome b556 subunit |  |
| **CE140_20030** | *sdhD* | Succinate dehydrogenase hydrophobic membrane anchor subunit |  |
| **CE140_21840** | *fumA* | Fumarate hydratase class I |  |
| **CE140_11695**  **CE140_15200** | *fumC* | Fumarate hydratase class II |  |
| **CE140_17540** | *maeB* | malate dehydrogenase (oxaloacetate-decarboxylating)(NADP+) |  |
| **CE140_06710**  **CE140_23200** | *mqo* | malate dehydrogenase (quinone) |  |
| **CE140_20040** | *gltA* | citrate synthase |  |
| **CE140_28170** | pycA | pyruvate carboxylase subunit A |  |
| **CE140_28165** | pycB | pyruvate carboxylase subunit B |  |
| **CE140_10625** | *acnA* | aconitate hydratase | **Glyoxylate bypass TCA** |
| **CE140_03220** | *acnB* | aconitate hydratase 2 / 2-methylisocitrate dehydratase |  |
| **CE140_01215** | *aceA* | isocitrate lyase |  |
| **CE140_25965** | *aceB* | malate synthase |  |
| **CE140_12440** | *aceK* | isocitrate dehydrogenase kinase/phosphatase |  |
| **CE140_03520**  **CE140_22880** | *ghrB* | Glyoxylate/hydroxypyruvate reductase B | **Glyoxylate degradation** |
| **CE140_05070** | *ghrA* | glyoxylate/hydroxypyruvatereductase |  |
| **CE140_04800**  **CE140_21000**  **CE140_23665** | *acs* | acetyl-CoA synthetase | **2-Methylcitrate Cycle, propanoate, oxaloacetate catabolism** |
| **CE140_19030** | *prpC* | 2-methylcitrate synthase |  |
| **CE140_19025** | *acnD* | 2-methylcitrate dehydratase (2-methyl-trans-aconitate forming) |  |
| **CE140_19020** | *prpF* | 2-methylaconitate isomerase |  |
| **CE140_03220** | *acnB* | aconitate hydratase 2 / 2- methylisocitrate dehydratase |  |
| **CE140_19035** | *prpB* | methylisocitrate lyase |  |
| **CE140_03000** | *fdsD* | formate dehydrogenase subunit delta | **Formate degradation** |
| **CE140_02995** | *fdoG* | formate dehydrogenase major subunit |  |
| **CE140_02990** | *fdoH* | formate dehydrogenase iron-sulfur subunit |  |
| **CE140_02985** | *fdoI* | formate dehydrogenase subunit gamma |  |
| **CE140_04800**  **CE140_21000**  **CE140_23665** | *acs* | Acetyl-coenzyme A synthetase | **Acetate degradation** |
| **CE140_26530** | *mdcD* | malonate decarboxylase beta subunit | **Malonate degradation I (biotin-independent)** |
| **CE140_26525** | *mdcE* | malonate decarboxylase gamma subunit |  |
| **CE140_05085** | *ttuC* | tartrate dehydrogenase/decarboxylase / D-malate dehydrogenase | **Tartrate degradation** |
| **CE140_15840** | *tarD* | D(-)-tartrate dehydratase |  |
| **CE140_08800** | *bdh* | 3-hydroxybutyrate dehydrogenase | **3-hydroxybutyrate degradation** |
| **CE140_05980**  **CE140_20045** | *actP* | cation/acetate symporter | **Acetate transport** |
| **CE140_06695**  **CE140_07530**  **CE140_07580**  **CE140_28875** | TC.CITMHS | citrate-Mg2+:H+ or citrate-Ca2+:H+ symporter, CitMHS family | **Citrate transport** |

**Table S12**- Genes involved in amino acid, peptides and opines metabolism and transport

| **Locus tag** | **Gene** | **Product** | **Pathway** |
| --- | --- | --- | --- |
| **CE140_26375** | *metK* | methionine adenosyltransferase | **Methionine degradation** |
| **CE140_14175** | *map* | Methionine aminopeptidase |  |
| **CE140_24675** | *mdeA* | L-methionine gamma-lyase |  |
| **CE140_08840** | *ridA* | 2-iminobutanoate/2-iminopropanoate deaminase |  |
| **CE140_05390**  **CE140_14680** | *ybdL* | methionine transaminase |  |
| **CE140_27610** | *alr* | alanine racemase | **D-alanine degradation** |
| **CE140_16315** | *speA* | arginine decarboxylase | **L-arginine degradation** |
| **CE140_09730**  **CE140_09735**  **CE140_20960**  **CE140_20965** | *astA* | Arginine N-succinyltransferase |  |
| **CE140_22355**  **CE140_22940** | *dauA* | D-arginine dehydrogenase | **D-arginine degradation** |
| **CE140_22350** | *dauB* | NAD(P)H-dependent anabolic L-arginine dehydrogenase DauB |  |
| **CE140_01945** | *aruH* | arginine:pyruvate transaminase |  |
| **CE140_00325**  **CE140_02765** | *aspB* | aspartate aminotransferase | **L-aspartate degradation/conversion** |
| **CE140_20920** | *ak* | aspartate kinase |  |
| **CE140_00415**  **CE140_22725** | *-* | Aspartate-semialdehyde dehydrogenase |  |
| **CE140_29180** | *aspA* | Aspartate ammonia-lyase |  |
| **CE140_23655** | *panD* | Aspartate 1-decarboxylase |  |
| **CE140_12720** | *nadB* | L-aspartate oxidase |  |
| **CE140_12460** | *cysK* | cysteine synthase A | **L-cysteine degradation** |
| **CE140_24960** | *cysM* | cysteine synthase B |  |
| **CE140_14760**  **CE140_18950** | *iscS* | cysteine desulfurase | **Cysteine transformation** |
| **CE140_14210** | *sufS* | cysteine desulfurase / selenocysteine lyase |  |
| **CE140_18340** | *dcyD* | D-cysteine desulfhydrase | **D-cysteine degradation** |
| **CE140_03555** | *gdhA* | glutamate dehydrogenase (NAD(P)+) | **L-glutamate degradation** |
| **CE140_11205**  **CE140_13240** | *alaA* | Glutamate-pyruvate aminotransferase |  |
| **CE140_00825** | *glsA* | Glutaminase | **L-glutamine degradation** |
| **CE140_17485** | *gltB* | glutamate synthase (NADPH/NADH) large chain |  |
| **CE140_17480** | *gltD* | glutamate synthase (NADPH/NADH) small chain |  |
| **CE140_17330**  **CE140_17715**  **CE140_17720** | *hutH* | histidine ammonia-lyase | **L-histidine degradation** |
| **CE140_02040** | *ilvE* | branched-chain amino acid aminotransferase | **L-leucine, L-isoleucine and L-phenylalanine degradation** |
| **CE140_17215** | *putA* | proline dehydrogenase | **L-proline degradation** |
| **CE140_21490**  **CE140_26225** | *sdaA* | L-serine dehydratase | **L-serine degradation** |
| **CE140_02630**  **CE140_04115**  **CE140_18445**  **CE140_22750** | *ilvA* | threonine dehydratase | **L-threonine degradation** |
| **CE140_01910**  **CE140_24165** | *hppD* | 4-hydroxyphenylpyruvate dioxygenase | **L-tyrosine degradation** |
| **CE140_16060** | *mmsB* | 3-hydroxyisobutyrate dehydrogenase | **L-valine degradation** |
| **CE140_11205** | *avtA* | valine--pyruvate aminotransferase |  |
| **CE140_23830** | *thiO* | glycine oxidase | **Glycine degradation** |
| **CE140_21485** | *gcvP* | glycine dehydrogenase |  |
| **CE140_15800**  **CE140_26485**  **CE140_27620** | *dadA* | D-amino-acid dehydrogenase | **D-amino acid degradation** |
| **CE140_05140** | dthadh | threo-3-hydroxy-D-aspartate ammonia-lyase | **Threo-3-hydroxy-D-aspartate degradation** |
| **CE140_05490**  **CE140_03845**  **CE140_09850** | *ooxA* | Opine oxidase subunit A | **Opine catbolism** |
| **CE140_05500** | *ooxB* | Opine oxidase subunit A |  |
| **CE140_02215** | *mocC* | Rhizopine catabolism protein MocC |  |
| **CE140_22335** | *aapJ* | general L-amino acid transport system substrate-binding protein | **General L-amino acid transporter** |
| **CE140_22330** | *aapQ* | general L-amino acid transport system permease protein |  |
| **CE140_22325** | *aapM* | general L-amino acid transport system permease protein |  |
| **CE140_07740**  **CE140_22320** | *aapP* | general L-amino acid transport system ATP-binding protein |  |
| **CE140_18355** | *ABC.CYST.A* | cystine transport system ATP-binding protein | **Cystine transporter** |
| **CE140_18350** | *ABC.CYST.P* | cystine transport system permease protein |  |
| **CE140_15825**  **CE140_18345**  **CE140_18400** | *fliY* | cystine transport system substrate-binding protein |  |
| **CE140_18380**  **CE140_18535**  **CE140_28720** | *metQ* | D-methionine transport system substrate-binding protein | **D-Methionine transporter** |
| **CE140_18390**  **CE140_28725** | *metI* | D-methionine transport system permease protein |  |
| **CE140_18385**  **CE140_28730** | *metN* | D-methionine transport system ATP-binding protein |  |
| **CE140_02515**  **CE140_13690** | *argT* | lysine/arginine/ornithine transport system substrate-binding protein | **Lysine/arginine/ornithine transporter** |
| **CE140_02505**  **CE140_13700** | *hisM* | histidine transport system permease protein | **Histidine transporter** |
| **CE140_02510**  **CE140_13695** | *hisQ* | histidine transport system permease protein |  |
| **CE140_13710** | *hisP* | histidine transport system ATP-binding protein |  |
| **CE140_05480**  **CE140_09680**  **CE140_20995**  **CE140_22365** | *aotJ* | arginine/ornithine transport system substrate-binding protein | **Arginine/ornithine transport** |
| **CE140_20985** | *aotM* | arginine/ornithine transport system permease protein |  |
| **CE140_20990** | *aotQ* | arginine/ornithine transport system permease protein |  |
| **CE140_20980** | *aotP* | arginine/ornithine transport system ATP-binding |  |
| **CE140_21425**  **CE140_26480** | *gltI* | glutamate/aspartate transport system substrate-binding protein | **Glutamate/aspartate transporter** |
| **CE140_21435** | *gltK* | glutamate/aspartate transport system permease protein |  |
| **CE140_21430** | *gltJ* | glutamate/aspartate transport system permease protein |  |
| **CE140_21440** | *gltL* | glutamate/aspartate transport system ATP-binding protein |  |
| **CE140_28845** | *gltP* | proton glutamate symport protein |  |
| **CE140_19920** | *-* | putative tryptophan/tyrosine transport system substrate-binding protein | **Tryptophan/tyrosine transport** |
| **CE140_18020** | *-* | putative lysine/arginine/ornithine/histidine/octopine transport system substrate-binding protein | **Lysine/arginine/ornithine/histidine/octopine/opine** **transport** |
| **CE140_18025** | *-* | putative lysine/arginine/ornithine/histidine/octopine transport system permease protein |  |
| **CE140_18030** | *-* | putative lysine/arginine/ornithine/histidine/octopine transport system permease protein |  |
| **CE140_18015** | *-* | putative lysine/arginine/ornithine/histidine/octopine transport system ATP-binding protein |  |
| **CE140_06985**  **CE140_07610**  **CE140_13520**  **CE140_16695** | *livK* | branched-chain amino acid transport system substrate-binding protein | **Branched-chain amino acid transporter** |
| **CE140_06995**  **CE140_07620**  **CE140_13525**  **CE140_16690** | *livH* | branched-chain amino acid transport system permease protein |  |
| **CE140_06990**  **CE140_07615**  **CE140_08425**  **CE140_13530**  **CE140_16685** | *livM* | branched-chain amino acid transport system permease protein |  |
| **CE140_07000**  **CE140_08425**  **CE140_13535**  **CE140_16680** | *livG* | branched-chain amino acid transport system ATP-binding protein |  |
| **CE140_07605**  **CE140_13540**  **CE140_16675** | *livF* | branched-chain amino acid transport system ATP-binding protein |  |
| **CE140_01595**  **CE140_02395**  **CE140_02405**  **CE140_02750**  **CE140_02755**  **CE140_04230**  **CE140_06750**  **CE140_07255**  **CE140_07575**  **CE140_07765**  **CE140_08860**  **CE140_10755**  **CE140_17910**  **CE140_18515**  **CE140_19360**  **CE140_22760**  **CE140_25110**  **CE140_26295**  **CE140_26440**  **CE140_27295**  **CE140_28145** | *ABC.PA.S* | polar amino acid transport system substrate-binding protein | **Polar amino acid transport** |
| **CE140_02770**  **CE140_02775**  **CE140_07250**  **CE140_07755**  **CE140_08850**  **CE140_15815**  **CE140_18525**  **CE140_18530**  **CE140_22765**  **CE140_22770**  **CE140_27300**  **CE140_27305**  **CE140_28150** | ABC.PA.P | polar amino acid transport system permease protein |  |
| **CE140_02780**  **CE140_07245**  **CE140_08845**  **CE140_15820**  **CE140_18520**  **CE140_27310** | ABC.PA.A | polar amino acid transport system ATP-binding protein |  |
| **CE140_15355**  **CE140_15360**  **CE140_15365**  **CE140_15375**  **CE140_27680** | *dppA* | dipeptide transport system substrate-binding protein | **Dipeptide transport** |
| **CE140_15380** | *dppB* | dipeptide transport system permease protein |  |
| **CE140_15385** | *dppC* | dipeptide transport system permease protein |  |
| **CE140_15390** | *dppD* | dipeptide transport system ATP-binding protein |  |
| **CE140_15395** | *dppF* | dipeptide transport system ATP-binding protein |  |
| **CE140_05115**  **CE140_07905**  **CE140_08145**  **CE140_14985**  **CE140_15955** | ABC.PE.S | peptide/nickel transport system substrate-binding protein | **Peptide/nickel transport** |
| **CE140_05110**  **CE140_07910**  **CE140_08140**  **CE140_14980**  **CE140_15935** | ABC.PE.P | peptide/nickel transport system permease protein |  |
| **CE140_05105**  **CE140_07915**  **CE140_08135**  **CE140_14975**  **CE140_15940** | ABC.PE.P1 | peptide/nickel transport system permease protein |  |
| **CE140_05100**  **CE140_07920**  **CE140_14970**  **CE140_15945**  **CE140_05100** | ABC.PE.A1 | peptide/nickel transport system ATP-binding protein |  |

**Table S13**- Genes involved in phenolics, lignin and flavonoids metabolism.

| **Locus tag** | **Gene** | **Product** | **Pathway** |
| --- | --- | --- | --- |
| **CE140_06595** | *fcs* | feruloyl-CoA synthase | **Catabolism of hydroxycinnamic acids** |
| **CE140_06600** | *vdh* | Vanillin dehydrogenase |  |
| **CE140_06605** | *hchL* | Hydroxycinnamoyl-CoA hydratase-lyase |  |
| **CE140_05510** | *vanA* | Vanillate O-demethylase oxygenase subunit | **vanillyl-alcohol degradation**  **3,4-dihydroxybenzoate** |
| **CE140_05515** | *vanB* | Vanillate O-demethylase oxidoreductase |  |
| **CE140_13495** | *pobA* | p-hydroxybenzoate 3-monooxygenase | **Hydroxybenzoate degradation via hydroxylation** |
| **CE140_05905**  **CE140_07865**  **CE140_13080**  **CE140_15285** | *pcaC* | 4-carboxymuconolactone decarboxylase | **3,4-dihydroxybenzoate Protocatechaute degradation via beta-ketoadipate** |
| **CE140_13085** | *pcaD* | 3-oxoadipate enol-lactonase |  |
| **CE140_13090** | *pcaB* | 3-carboxy-cis,cis-muconate cycloisomerase |  |
| **CE140_13100** | *pcaG* | protocatechuate 3,4-dioxygenase subunit alpha |  |
| **CE140_13105** | *pcaH* | protocatechuate 3,4-dioxygenase subunit beta |  |
| **CE140_13110** | *pcaF* | 3-oxoadipyl-CoA thiolase |  |
| **CE140_13115** | *pcaJ* | 3-oxoadipate CoA-transferase subunit B |  |
| **CE140_13120** | *pcaI* | 3-oxoadipate CoA-transferase subunit A |  |
| **CE140_07275** | *mhpA* | 3-(3-hydroxy-phenyl)propionate hydroxylase | **3-phenylpropanoate degradation** |
| **CE140_06065**  **CE140_06070** | *hpaG* | 5-oxopent-3-ene-1,2,5-tricarboxylate decarboxylase /  2-hydroxyhepta-2,4-diene-1,7-dioate isomerase | **4-hydroxyphenylacetate degradation, p-coumaric acid degradation** |
| **CE140_06075** | *hpaE* | 5-carboxymethyl-2-hydroxymuconic-semialdehyde dehydrogenase |  |
| **CE140_06080** | *hpaD* | 3,4-dihydroxyphenylacetate 2,3-dioxygenase |  |
| **CE140_06085**  **CE140_12580** | *hpaF* | 5-carboxymethyl-2-hydroxymuconate isomerase |  |
| **CE140_06095** | *hpaH* | 2-oxo-hept-3-ene-1,7-dioate hydratase |  |
| **CE140_04610**  **CE140_06100** | *hpaI* | 4-hydroxy-2-oxoheptanedioate |  |
| **CE140_06110** | *C1-hpah* | p-hydroxyphenylacetate 3-hydroxylase reductase component |  |
| **CE140_06115** | *C2-hpah* | p-hydroxyphenylacetate 3-hydroxylase oxygenase component |  |
| **CE140_10740**  **CE140_19845** | *curA* | NADPH-dependent curcumin reductase | **Curcumin metabolism** |
| **CE140_09525** | *-* | Dyp type peroxidase | **Lignin metabolism** |
| **CE140_23790** | *yfiH* | polyphenol oxidase/laccase |  |
| **CE140_06645** | *calA* | Coniferyl-alcohol dehydrogenase | **Monolignol metabolism** |
| **CE140_06650** | *calB* | coniferyl-aldehyde dehydrogenase |  |
| **CE140_05035** | *dad* | 2,4′-dihydroxyacetophenone dioxygenase | **2,4′-dihydroxyacetophenone catabolism** |
| **CE140_07290** | *catE* | catechol 2,3-dioxygenase | **Catechol metabolism** |
| **CE140_08155-08295** | *fde gene cluster* | Naringenin degradation cluster | **Flavonoid metabolism** |
| **CE140_00190**  **CE140_01090**  **CE140_04475** | *pir* | quercetin 2,3-dioxygenase |  |

**Table S14-** Genes involved in phytohormone modulation.

| **Locus tag** | **Gene** | **Product** | **Pathway** |
| --- | --- | --- | --- |
| **CE140_07955** | *acdS* | 1-aminocyclopropane-1-carboxylate deaminase | **ACC degradation** |
| **CE140_07960** | *acdR* | AsnC family transcriptional regulator |  |
| **CE140_07340** | *tlpQ* | Chemotaxis protein | **ET receptor MCP** |
| **CE140_25830** | *iaaM* | Tryptophan 2-monooxygenase | **IAA biosynthesis**  **IAM** |
| **CE140_02695**  **CE140_06190** | *amiE* | amidase |  |
| **CE140_14675** | *yafV* | omega-amidase |  |
| **CE140_06180**  **CE140_07305** | *feaB* | phenylacetaldehyde dehydrogenase | **PAA biosynthesis** |
| **CE140_16865** | *miaA* | tRNA dimethylallyltransferase | **CK biosynthesis/modificatios** |
| **CE140_24365** | *miaB* | tRNA (N6-isopentenyl adenosine(37)-C2)-methylthiotransferase |  |
| **CE140_03235** | *miaE* | tRNA-(ms[2]io[6]A)-hydroxylase |  |
| **CE140_13860**  **CE140_16740** | *log* | cytokinin riboside 5'-monophosphate phosphoribohydrolase |  |
| **CE140_10800** | *xdhA* | xanthine dehydrogenase small subunit |  |
| **CE140_10805** | *xdhB* | xanthine dehydrogenase large subunit |  |
| **CE140_10810** | *xdhC* | xanthine dehydrogenase accessory protein XdhC |  |
| **CE140_24105** | *yagR* | xanthine dehydrogenase YagR molybdenum-binding subunit |  |
| **CE140_24110** | *yagS* | xanthine dehydrogenase YagS FAD-binding subunit |  |
| **CE140_24115** | *yagT* | xanthine dehydrogenase YagT iron-sulfur-binding subunit |  |
| **CE140_05240** | *pchA* | isochorismate synthase | **SA biosynthesis** |
| **CE140_03015**  **CE140_05235** | *pchB* | isochorismate pyruvate lyase |  |

**Table S15**- Genes involved in the biosynthesis of antagonistic traits

| **Locus tag** | **Gene** | **Product** | **Pathway** |
| --- | --- | --- | --- |
| **CE140_07395** | *phlH* | transcriptional regulator of 2,4-DAPG biosynthesis | **2,4-diacetylphloroglucinol** (**DAPG) biosynthesis** |
| **CE140_07395** | *phlG* | 2,4-diacetylphloroglucinol hydrolase |  |
| **CE140_07400** | *phlF* | TetR family transcriptional regulator/  PhlF transcriptional repressor |  |
| **CE140_07405** | *phlA* | hydroxymethylglutaryl-CoA synthase |  |
| **CE140_07410** | *phlC* | acetyl-CoA C-acetyltransferase  2,4-diacetylphloroglucinol biosynthesis protein |  |
| **CE140_07415** | *phlB* | 2,4-diacetylphloroglucinol biosynthesis protein |  |
| **CE140_07420** | *phlD* | type III polyketide synthase |  |
| **CE140_07425** | *phlE* | MFS transporter |  |
| **CE140_02185** | *hcnA* | Hydrogen cyanide synthase subunit HcnA | **Hydrogen cyanide**  **biosynthesis** |
| **CE140_02190** | *hcnB* | Hydrogen cyanide synthase subunit HcnB |  |
| **CE140_02195** | *hcnC* | Hydrogen cyanide synthase subunit HcnC |  |
| **CE140_03710-15** | Bacteriocin cluster | Bacteriocin biosynthesis | **Bacteriocin production** |
| **CE140_13550-70** | Bacteriocin cluster | Bacteriocin biosynthesis |  |
| **CE140_18560** | Bacteriocin cluster | Bacteriocin biosynthesis |  |
| **CE140_23990** | Lantipeptide cluster | Lantipeptide biosynthesis | **Lantipeptide production** |
| **CE140_13675**  **CE140_27020** | *quiP* | Acyl-homoserine lactone acylase QuiP | **Quorum-quenching** |
| **CE140_05540** | *pvdQ* | Acyl-homoserine lactone acylase PvdQ |  |
